# Systematic mapping review of statistical methods applied to the relationships between cancer diagnosis and geographical level factors in UK

**DOI:** 10.1136/bmjopen-2024-098379

**Published:** 2025-07-06

**Authors:** Jessica Andretta Mendes, Thomas Keegan, Lisa Jones, Peter M Atkinson, Luigi Sedda

**Affiliations:** 1 Lancaster University Medical School, Lancaster, UK; 2 Nuffield Department of Medicine, University of Oxford, Oxford, UK; 3 Liverpool John Moores University Faculty of Health, Liverpool, UK; 4 Lancaster Environment Centre, Lancaster University, Lancaster, UK

**Keywords:** ONCOLOGY, Epidemiology, Review, Risk Factors, STATISTICS & RESEARCH METHODS

## Abstract

**Abstract:**

**Objectives:**

We examined studies that analysed the spatial association of cancers with demographic, environmental, behavioural and/or socioeconomic factors and the statistical methods applied.

**Design:**

Systematic mapping review.

**Data sources:**

Web of Science (SSCI) (search on 28 July 2022), MEDLINE, SocINDEX and CINAHL (search on 4 August 2022), additional searches included grey literature.

**Eligibility criteria for selecting studies:**

(1) Focused on the constituent countries of the UK (England, Wales, Scotland and Northern Ireland) and its major regions (eg, the North West); (2) compared cancer(s) outcomes with demographic, environmental, behavioural and socioeconomic characteristics by applying methods to identify their spatial association; (3) reported cancer prevalence, incidence rates, relative risk or ORs for a risk factor or to an average level of cancer.

**Data extraction and synthesis:**

A standardised data extraction form was developed and for all studies, core data were extracted including bibliographic information, study design, geographical factors analysed, data aggregation level, methods applied and main findings. We described and synthesised the characteristics of the studies using summary tables, charts and graphs.

**Results:**

52 studies were included covering a variety of objectives and geographical scales. These studies considered different types of cancer, with the most common cancer types analysed being blood and lymphoid cell cancers. The most common methods used to assess the association between cancers and geographical level factors were regression analyses, with the majority being Poisson regression, then logistic and linear regression. Studies were usually conducted at ward and local authority level, or by exact point location when distances from putative risk sources were considered. The results were usually presented in plots or as tables, instead of maps.

**Conclusion:**

Our results highlight the lack of consideration of spatially explicit models in the analysed studies, with the risk of having failed the assumption of independence in the data.

**PROSPERO registration number:**

CRD42022349165.

**STRENGTHS AND LIMITATIONS OF THIS STUDY**

- A comprehensive search strategy was applied (four different databases consulted, encompassing terms related to different types of cancers, including socioeconomic, demographic, behaviour and environmental factors).
- A quality assessment was conducted for each eligible study.
- To retrieve as many studies as possible that could be assessed, our searches had no restrictions regarding literature, date of publication or language, and we configured to receive alerts for a longer period.
- A single-screener approach has been applied.
- Due to heterogeneity in study designs and methods of our included studies, we did not extract numerical results for most of them.

## Introduction

 Globally, cancer is among the leading causes of mortality. Trachea, bronchus and lung cancer deaths are estimated to have risen from 1.2 million in 2000 to 1.9 million in 2021.^1^ The most common types of cancer in terms of incidence are breast, lung, colon and rectal, prostate, skin (non-melanoma) and stomach cancer. The early detection of these and other types of cancer increases the likelihood of positive treatment outcomes and less expensive treatments.^2^

Lifestyle and individual behavioural factors can impact significantly on cancer risk.^3 4^ However, research suggests that cancers are not attributable solely to individual lifestyle and behavioural factors or result from individual preferences and decision-making, but are also influenced by genetics and structural and environmental determinants^5^ (or geographical level factors) such as neighbourhood poverty level, features of the built environment and access to health care.^5 6^

Studies report that cancer incidence and their determinants, including geographical level factors, are likely to be affected by spatial dependence.^7^,^9^ In this case, spatially explicit models must be considered, as they can incorporate spatial dependence and spatial heterogeneity into the analysis, providing more accurate estimates of cancer risk.^7^,^9^ In fact, the geographical distribution of cancer cases may exhibit spatial clustering patterns partly or fully dependent on factors like socioeconomic status (SES) or unknown risk factors common to other diseases.^8^ Not accounting for the unexplained spatial dependence in the cancer distribution may lead to biased estimates and misleading conclusions about relationships and associations between variables. In these cases, spatial epidemiology can contribute to identifying spatial patterns and differences in disease burden across areas through mapping and clustering detection.^10^ Using these methods, disease mapping studies can identify the spatial variation in disease risk and highlight areas of elevated or lowered risk that can provide clues to the disease aetiology. Downing *et al*^11^ highlighted that disease mapping provides useful background information for researchers, the government and the public, especially in the planning of services or in cancer prevention and control programmes.

We conducted a systematic mapping review of studies that explored the geographical level impact of demographic, environmental, behavioural and/or socioeconomic factors on cancer diagnosis (in broad terms, eg, stage, delay, uptake) and incidence. By framing this as an analysis using *geographical level factors*, we focus our interest on studies that analysed the spatial variation in cancers and its association with spatially varying demographic, environmental, behavioural and/or socioeconomic factors.

Of specific interest for this review are the methods used to evaluate the spatial distribution of geographical level factors associated with cancers, including co-occurrence and joint mapping approaches. Many diseases share common risk factors and, recently, techniques such as joint disease mapping have exploited this property to refine estimates, such as incidence rates, and provide estimation even in areas where cancer incidence information is not available.^11^

We focused this research on UK studies and, in particular, on the North West of England. This region has significantly higher overall cancer incidence rates compared with the national average, with certain areas such as Liverpool and Manchester experiencing 10–15% more deaths from cancer than the national average for England.^12^ There is also an established difference in health between the North and South of England, which has its roots in the industrial revolution and more recent uneven economic development.^13^ Variation in opportunities to access programmes that can lead to improved health, such as early years education, as well as differences in economic and food security, play key roles in maintaining these regional health differences.^13^

This review assessed the studies’ objectives, the level of aggregation of data in the analyses and the types of cancers considered; we paid special attention to the methods employed to analyse the geographical patterns of cancers and the selected potential risk factors in studies from the UK.

## Material and methods

### Mapping and analysis

This paper constitutes a systematic mapping review. In fact, the heterogeneity between our included studies does not support a quantitative analysis.^14^ We considered that a statistical synthesis required a sufficient level of homogeneity across studies in terms of population, intervention, outcome measures and methodological approaches. In contrast, the studies included in this review exhibit substantial diversity across multiple dimensions including study designs, objectives, data collected in different time periods, population characteristics (age groups, sex), spatial scales (eg, from Lower Layer Super Output Area - LSOA - to country level), outcome measures (eg, incidence rates, ORs, risk ratios), covariate adjustments and methodological approaches. Therefore, we considered that a narrative synthesis would be more appropriate for summarising the existing evidence.

### Protocol preparation and preliminary search

Prior to protocol preparation, we searched for systematic reviews on the International Prospective Register of Systematic Reviews, PROSPERO, that considered the geographical distribution of different types of cancers in the UK and the potential risk factors such as socioeconomic, environmental and/or behavioural factors. None was found, so we drafted a protocol for conducting systematic reviews of observational studies of aetiology based on that provided by COSMOS-E (conducting systematic reviews and meta-analyses of observational studies of aetiology).^15^ This study was registered as a systematic review on PROSPERO (Registration number: CRD42022349165).

We tested different search strategies, using the MEDLINE database. We performed searches iteratively until no new titles were identified and retrieved all relevant studies from the search strategy tests.^16^,^21^

### Search strategy and study selection

The inclusion and exclusion criteria for studies are detailed in the online supplemental file 1.

**Figure 1:**
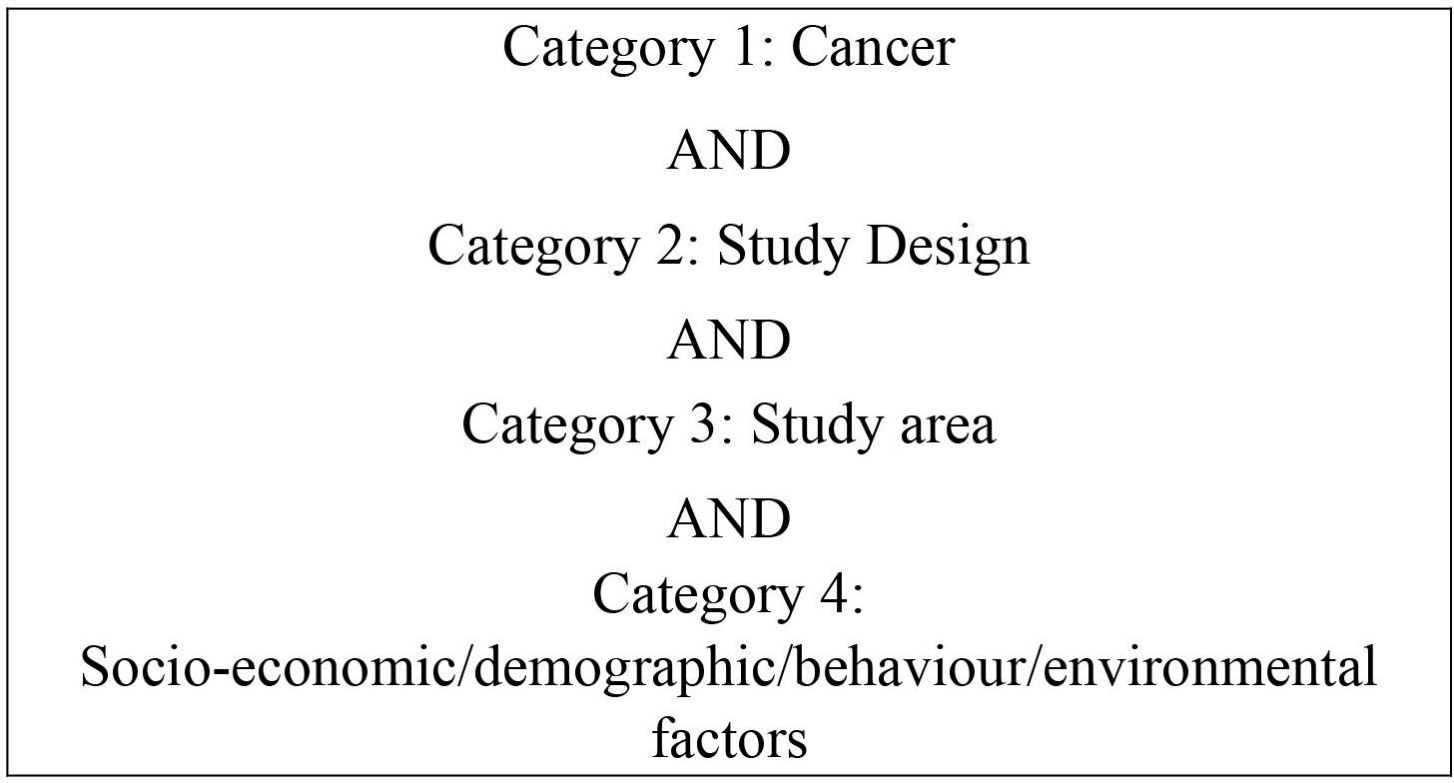
Categories of terms used in the search strategy.

We conducted searches in four databases: Web of Science Social Sciences Citation Index—SSCI (in 28 July 2022), MEDLINE, SocINDEX and CINAHL (in 4 August 2022), with no restriction regarding literature, date of publication or language. We searched for terms in the title, abstract and controlled vocabulary (eg, Medical Subject Headings) (search terms provided in online supplemental file 2). To outline, the search terms were grouped as per the following exemplar search strategy (figure 1):

We also conducted handsearching (searching through journals or conference publications which are not indexed in the major electronic databases) and searched in the grey literature to complement our database searches. We searched for conference papers published in the 19th International Medical Geography Symposium, reports from North West Cancer Research and in the reference lists of the retrieved papers. In addition, between 13 May 2023 and 31 March 2024, we configured to receive alerts through the Scopus database.

### Data extraction and quality appraisal

A standardised data extraction form was developed and piloted on a sample of studies. For all studies, core data were extracted, including bibliographic information, study design, geographical factors analysed, data aggregation level, methods applied and main findings (online supplemental file 3). Where a paper discussed aspects other than the association between cancer and potential risks, such as survival and/or mortality, we focused on retrieving information on cancer diagnosis, incidence or prevalence and did not extract data on the other factors.

The studies employed a variety of methods depending on the topic and aims of their research. We concentrated on extracting information on the methods used for spatial/temporal analysis of cancers and potential risk factors, or those which analysed the association between the two. We did not extract numerical results for most of our included studies. As an example, online supplemental file 4 illustrates the heterogeneity of numerical results for studies applying spatially explicit methods.

Retrieved studies reported different cancer types, sites and subsites, including different versions for International Classification of Diseases (ICD) codes. To synthesise and analyse the included studies, cancer types were aggregated into categories to facilitate the descriptive analysis (eg, blood and lymphoid cells encompassing leukaemia, lymphoma, lymphatic haematological, myelomas, Hodgkin lymphoma and non-Hodgkin’s lymphoma; liver cancer encompassing hepatocellular carcinoma and angiosarcoma of the liver; central nervous system tumours and neural tumours encompassing gliomas, glioblastoma, neuroblastoma and ganglioneuroblastoma; skin cancers encompassing non-melanoma and melanoma). We used as reference the categories for cancer types and tumour lists presented by institutions like UK Cancer Research (https://www.cancerresearchuk.org/about-cancer/type) and the National Cancer Institute (https://www.cancer.gov/types).

For each eligible study, an assessment of quality was conducted (online supplemental files 5 and 6). Since different types of study design were included in this systematic mapping review, we used two different assessment tools: the Newcastle-Ottawa Quality Assessment for case-control studies;^22^ and the Joanna Briggs Institute (JBI) Checklist for Prevalence/Incidence Studies, which was also used to assess cohort studies reporting prevalence/incidence.^23^ To retain consistency between different study designs, we omitted scoring. The quality assessments were used only for descriptive reporting. We focused on systematically mapping and describing the extent of the literature available, and it is not part of the scope of this work to compile quantitative information for assessment (eg, in providing a relative risk or prevalence of cancers based on location or correlation between cancers).

## Results

The searches retrieved over 10 000 results: 4583 from the Web of Science/SSCI; 4672 from Medline; 40 from SocINDEX; and 915 from CINAHL database (online supplemental file 7). We removed duplicates in EndNote as recommended by Bramer *et al*.^24^ Titles and abstracts of the remaining articles were screened using Rayyan, a research tool to import and manage articles for systematic reviews,^25^ and considering the inclusion/exclusion criteria described in the section on material and methods. Since this mapping review can be assimilated to a rapid review, a single screener approach has been employed.^26^

We selected 62 studies that could potentially be included in our review (online supplemental file 7). For six papers, the full text article was not available, and they were excluded. Four further papers were excluded after full text screening because their focus did not meet the inclusion criteria.

### Included studies general characteristics

The search resulted in a final selection of 52 studies from 26 journals. Of these, two were short reports,^27 28^ the remainder were full articles. 31 were ecological studies,^1117 27^,^55^ 8 were case-control studies^56^,^63^ and 7 were cohort studies.^64^,^70^ The remaining six studies^2071^,^75^ were classified according to the respective authors’ definitions, as descriptive epidemiological studies, longitudinal studies, population-based studies and cross-sectional studies (online supplemental files 8 and 9).

The earliest publication was from 1985 (online supplemental file 10). Between 1985 and 1995, 13 publications were included.^30^,^3856 57 64 71^ There were 16 publications published between 1996 and 2006,^2729 39^,^47 55 58^ and 16 between 2007 and 2017.^1117 28 48^,^53 61^ From 2018 onwards, we identified seven publications^2054 67^,^70 74^ (online supplemental files 9 and 10). These studies each had different study periods, with the earliest dating to 1945.^71^

### Objectives of the studies

There was variability in the research objectives, cancer types and potential risk factors, and the scale of analysis (online supplemental file 9). We identified two types of geographical approaches in these studies: the first, comprising 28 studies, analysed the spatial distribution and time-trends of disease and explored the relationship with geographical factors (eg, demographic, environmental, behaviour and socioeconomic factors);^1117 20 29 30 33 36 43 45 47^,^55 64 66^ the second focused on assessing the influence of proximity to putative risk sources (eg, heavy industry, nuclear installations, high-voltage powerlines) and comprised 24 studies^2728 31 32 34 35 37^,^42 44 46 56^ (online supplemental file 9).

### Study areas and level of aggregation

The included studies covered different geographical areas subdivided into geographical units of different scales, depending on the respective outcome of interest (online supplemental file 11).

Of all the included studies, 12 analysed cancer data using different geographical units within the same study and were classified as multilevel analysis (online supplemental files 9 and 11); in 22 studies, the data were aggregated into electoral wards, grid references, exposure zones, postcode districts, primary care trusts and general practices. Finally, six studies considered only the distance from prespecified potential risk source(s) (eg, radii). Online supplemental file 11 provides detailed information on the number of studies according to the geographical aggregation level of the data.

Most studies (n*=*29) were carried out only in England^1117 20 27 30 31 33^,^35 40 42 43 45 47^ and 6 in Great Britain^29 39 41 58 61 63^ (online supplemental file 12). Details of the geographical area covered in each study are provided in online supplemental file 9. Among those studies conducted within England, 10 studies contained specific analyses for the North West^20 30 33 34 43 45 51 53 56 57^ at the county, local authority or ward level (online supplemental file 9). Most of the North West studies were published between 1985 and 1995 (n=5),^30 33 34 56 57^ although two were published between 1996 and 2006,^43 45^ two between 2007 and 2017^51 53^ and one after 2018.^20^ The majority were ecological studies (n=7);^30 33 34 43 45 51 53^ two were case-control^56 57^ and one was a longitudinal study.^20^

The majority of the studies that focused on the North West of England investigated blood and lymphoid cell cancers (table 1).

**Table 1:** Types of cancers analysed in studies focusing on the North West of England

Number of studies	Study reference	Types of cancers studied
7	^30 33 34 43 45 56 57^	Blood and lymphoid cells
2	^33 34^	Brain tumours
1	^51^	Central nervous system tumours
1	^53^	Thyroid
1	^20^	Head and neck
1	^34^	Not specified

Within the studies that focused on the North West of England, demographic factors, including sex and age, and environmental factors, such as proximity to nuclear installations or overhead high-voltage powerlines (online supplemental file 13), were the main factors of interest. The analytical methods used varied, but the most common was regression analysis, in particular, Poisson, linear or logistic regression (table 2).

**Table 2:** Statistical methods used in studies focused on the North West of England

Number of studies	Study reference	Methods
4	^33 43 45 51^	Regression models (Poisson)
1	^43^	Regression models (linear)
1	^56^	Regression models (logistic)
2	^34 53^	Incidence rates comparison (study areas × national data)
2	^30 57^	Cluster analysis (Knox’s test for space–time interaction; Cuzick-Edwards test)
1	^20^	Correlation analysis (Pearson’s correlation)
1	^51^	Examine the significance of association (χ² tests)
1	^57^	Nearest neighbour computation

### Type of cancer

More than 20 cancer types were identified in all included articles (tables1  3 and online supplemental file 9). Because one article could address more than one cancer type, the number of total articles exceeds 52. Some studies referred to the category ‘others’, which might be associated with unspecified sites and indicated with ‘not specified (others)’ type of cancer (table 3). Most articles (n=32) refer to a single cancer type,^2027^,^32 37 41 43 45 47 49^ and the remainder refer to two or more types.

**Table 3:** Cancer types assessed in studies

Cancer types	Study reference	Number of studies
Blood and lymphoid cells	2728 30 35 37	27
Brain tumours	^33 34 36 39 40 42 44 46 59 62 63 65^	12
Skin	^33 34 36 39 40 42 44 46 59 62 63 65^	11
Lung	^11 17 35 40 46 55 60 69 74^	9
Central nervous system (CNS tumours, including gliomas, glioblastomas, neuroblastoma and ganglioneuroblastoma)	^36 40 51 52 59 62 63 65^	8
Breast	^17 40 46 47 50 62 65^	7
Not specified (others)	^34 35 58 59 63 65^	6
Colon and rectal	^40 46 68 74^	4
Thyroid	^46 53 64 65^	4
Stomach	^11 40 42 46^	4
Bladder	^11 39 40^	3
Liver	^41 54 70^	3
Other respiratory cancers (malignant neoplasms of respiratory and intrathoracic organs, upper respiratory tract tumours and lower respiratory tract tumours)	^42 46 65^	3
Genitourinary (malignant neoplasm of genitourinary organs and genitourinary tract tumours)	^35 65^	2
Oesophagus	^11 46^	2
Pancreas	^11 42^	2
Digestive cancer (malignant neoplasm of digestive organs and peritoneum) and gastrointestinal tract tumours	^35 65^	2
Eye (including choroid, ciliary body and iris)	^40 48^	2
Oral cancer (including mouth and lips)	^35 72^	2
Cervix and uterus	^17^	1
Head and neck	^20^	1
Kidney	^11^	1
Laryngeal	^38^	1
Prostate	^40^	1
Ovarian	^74^	1
Pharynx	^35^	1
Soft tissue sarcoma	^46^	1
Testicular	^29^	1

27 studies included analyses on blood and lymphoid cell cancers,^2728 30^,^35 37^ 12 brain tumours^33 34 36 39 40 42 44 46 59 62 63 65^ and 11 skin cancers.^17 35 39 40 42 48 49 62 65 66 73^ Among the blood and lymphoid cell cancers, the most frequently studied were leukaemia and non-Hodgkin’s lymphoma (online supplemental file 9).

### Potential risk factors

The common potential risk factors were those related to socioeconomic, demographic and environmental factors (online supplemental files 9 and 14).

Environmental factors included living in urban areas, exposure to sunshine, exposure to radon concentrations and proximity to putative risk sources (such as nuclear sites or high voltage powerlines). Socioeconomic factors were related to deprivation and SES, while demographic factors included age and sex. It is important to emphasise that the factors associated with cancer varied according to the studied population, cancer type and geographical scale. Lifestyle, behaviour and other elements were less explored in the included studies.

### Methods applied

A variety of methods were used to evaluate cancer and geographical level potential risk factors (table 4 and online supplemental file 9), including joint mapping approach to assess cancer co-occurrence. The most common were regression models (n=30 studies),^1129 31^,^33 35 37 43^ in particular Poisson (n=13 studies), logistic (n=10 studies) and linear (n=4 studies). Two studies employed general linear models for joint disease modelling within Bayesian frameworks.^11 55^ The remainder of the studies involved crude and standardised ratios and relative risk (n=13 studies).^17 27 28 37 39 40 42 46 52 65 66 71 75^

**Table 4:** The methods most applied to analyse cancers and their geographical level factors

Number of studies	Study reference	Methods applied
30	1129 31 33 35 37 43	Regression models
10	^17 27 28 37 39 40 42 46 65 71^	Observed and expected calculations
6	^17 28 52 65 66 75^	Incidence estimation
5	38 4042 44	Stone’s test
4	^20 47 55 62^	Correlation analysis
3	^34 53 65^	Incidence rates comparisons
2	^30 57^	Cluster analysis
1	^40^	Comparing differences between groups
1	^17^	Estimation of additional/fewer cases
1	^36^	Percentage change in incidence
1	^51^	Examining significance of associations
1	^48^	Estimation of admission rates
1	^57^	Nearest neighbour computation
1	^41^	Number of cases in each range distance from the potential risk site
1	^64^	OR calculation
1	^17^	Rate ratios

Regarding the presentation of the main results, those using maps accounted for 15 studies (29%),^1117 20 29 36 42 47^,^49 54 55 64 68 73 75^ while the majority of the included studies presented results using tables and/or plots (online supplemental file 9). For those 28 studies analysing the spatial distribution and time-trends of cancers, and exploring the relationship with geographical factors, we presented an outline of the context in which the methods were applied, along with a description of the strengths and limitations highlighted by the respective studies’ authors (online supplemental files 15–18).

### Quality appraisal of included studies

These checklists were used only for descriptive reporting. We evaluated eight case-control studies using the Newcastle-Ottawa appraisal tool.^56^,^63^ In terms of defining and representing cases, most studies employed record linkage to obtain data from data sets (eg, using ICD codes in database, from regional or national database), encompassing all eligible cases with the relevant outcome within a specified time frame. This was done within a defined catchment area, hospital or clinic, group of hospitals or through an appropriate sample of these cases (such as a random sample) (online supplemental files 5 and 6).

Regarding control selection and definition, most studies used community controls, selected from the same community as the cases. These controls were explicitly confirmed to have no history of outcome. Secure records were used, such as medical records or registry data. Half of the studies applied the same method to identify both cases and controls.^5661^,^63^ Five studies did not consider the participant’s involvement, so there was no assessment for response rates, and the remaining studies provided descriptions of non-respondents.^57 58 60^ All eight studies adjusted their analyses for age and other controlled variables (online supplemental files 5 and 6).

We assessed 44 studies using the JBI Checklist for Prevalence/Incidence Studies, similarly as performed by Soualhi *et al*.^76^ 40 studies used data from secure sources like registries, medical records and large national data sets.^1117 20 27 28 30^,^46 48^ The nature of the data allowed for adequate sample sizes and sufficient coverage. The same studies detailed their subjects and settings, demographic, socioeconomic and geographical variables and the statistical approaches used (online supplemental files 5 and 6).

The majority of the studies assessed using the JBI Checklist used quantitative approaches (n=41), relying on registries of cancer diagnosis data sets. Three studies^35 67 69^ (online supplemental files 5 and 6) were qualitative involving the recruitment of participants, surveys or questionnaires, which include, for example, large cohorts where participants were involved in discussions or where the study used self-reported data. For these studies, the response rate was adequate or managed appropriately. Most studies employed valid methods to assess outcomes based on existing definitions or diagnostic criteria (n=42),^1117 20 27^,^34 36^ while for two studies this was unclear.^35 55^ Here we are looking for measurement or classification bias regarding the methods used for the identification of the condition. We are considering whether the outcomes were assessed based on existing definitions or diagnostic criteria or if the outcomes were assessed using self-reported scales (in which objectivity is compromised and the risk of over-reporting or under-reporting is increased). Most of the studies assessed with this tool used suitable statistical analyses (n=40),^1117 20 27^,^34 36^ while for four studies this was unclear (online supplemental files 5 and 6). Despite the methods applied being suitable for the data, this assessment does not evaluate the justification of these methods or their potential for improvement.

## Discussion

This review examined studies of cancers (diagnosis and incidence) and their association with geographical level factors in the UK. With a broad scope, we built a comprehensive search strategy, which retrieved key papers for potential inclusion in our review. We identified gaps in the research literature and characterised and described the included studies. However, it is important to note that studies may have been missed due to the choice of search terms and the inclusion/exclusion criteria and by the fact that we used a single screener approach.

We acknowledge that due to the heterogeneity in study designs and methodologies across the studies we included, we were unable to extract numerical results for most of them. As a result, we opted for a narrative synthesis rather than conducting a statistical summary or quantitative analysis. To highlight this heterogeneity, we have provided numerical results for studies using spatially explicit methods (online supplemental file 4), covering both past and recent research on single or multiple cancer types.^11 20 29 30 45 47 51 55^ These studies differ in cancer types considered, age groups, period, geographical location and scale, methods and outcomes.

We selected studies that analysed the spatial association of cancers with demographic, environmental and/or socioeconomic factors, and of these studies, we examined the methods used to evaluate the spatial distribution of cancers and their association with geographical level factors, including co-occurrence/joint mapping approaches.

The studies retrieved in our initial search (>10 000) were focused primarily on genetic/biological conditions, screening/detection/diagnosis/surgical procedures, clinical outcomes, medication and treatments, management or experience of care and assessing the impact of awareness activities and campaigns, among others. The 52 studies retained in our review addressed more than 20 cancer types. For this reason, numerous associations were reported between different types of cancers and potential risk factors. The strengths of these associations also differed, likely due to variation in cancer types and in the outcomes being studied, and differences in the study populations, geographical region, geographical scale and the variables considered, similarly to Gomez *et al*.^6^

In our systematic mapping review, we observed that the majority of included studies applied Poisson and logistic regression, and few studies addressed the spatial dependence and spatial relationships in cancers and geographical level factors,^11 29 30 47 51 55 57^ which involve identifying clusters and evaluating the spatial dependence or autocorrelation within the data. As outlined in our introduction, by integrating spatial relationships and employing analytical techniques that acknowledge the spatial arrangement of data, we can more accurately locate spatial clusters and hotspots of disease incidence, which may help to uncover potential risk factors specific to particular geographical areas.^8^

Other reviews found similar results regarding the lack of research about spatial dependence and spatial relationships in studies assessing cancer outcomes and geographical level factors, such as neighbourhood characteristics or SES. Gomez *et al*^6^ conducted a review in which the objective was to identify articles in which specific social and/or built environment neighbourhood factors were investigated to be associated with outcomes across the cancer continuum (incidence, diagnosis, treatment, survivorship and survival).

In line with our findings, Gomez *et al*^6^ highlighted the need to incorporate concepts of space (such as proximity) in investigations of neighbourhood health effects. They also stressed that spatial analytic methods are important for assessing both neighbourhood exposure and spatial autocorrelation. Similarly, a comprehensive review conducted by Mihor *et al*^77^ on 91 studies indicated the scarcity of spatially explicit approaches. They revealed a diverse range of statistical methods and scales, both at individual and area level, but only two studies used spatial models (Besag York Mollié Model - BYM) in research on the relationship between cancer incidence and SES in Europe.

For the 28 studies analysing the spatial distribution and time-trends of cancers, while exploring the relationship with geographical factors, we noticed that, with few exceptions,^11 55^ the majority of the authors do not provide a comprehensive description of the statistical methods used, or a clear justification for their use. In discussions of strengths and limitations, authors tended to highlight aspects regarding the data set (data quality, completeness or accuracy; availability of cancer subtypes or patients’ outcomes), or aspects regarding time coverage or potential inclusion of new covariates. Few studies described strengths and limitations regarding the statistical methods used.^11 20 28 29 31 45 49 64 74^ The most recent studies (those published from 2010 onwards) mostly considered one cancer type and usually used regression analysis to assess any association with covariates. Only two recent studies considered multiple cancer types.^48 74^ We also noticed that only a few recent studies used spatially explicit approaches, such as Sehmer *et al*.^51^ A joint modelling approach was found only in studies published in 2006 and 2008^11 55^ (online supplemental files 15–18). Even though other studies assessed multiple cancer types together (see, eg, Keenan *et al*,^48^ Conway *et al*^72^ and Muller *et al*),^74^ the authors did not provide any justification for the absence of data modelling, or for the use of aspatial models. Neither did they provide discussions about the potential presence of spatial dependence nor consider the improvement of their methods by using a joint approach.

Another systematic review conducted by Arcaya *et al*^78^ analysed studies in the USA and included 256 articles with diverse outcomes related to health and health behaviours, ranging from mental health, anthropometric measures, cancer and cardiovascular health. The authors highlighted that in the vast majority of articles included, spatial relationships among neighbourhood units were not considered, nor were neighbourhoods situated within larger geographies (eg, by including supra-neighbourhood characteristics like the municipal policy environment, which shapes neighbourhoods through services, policies and programmes). They argue that excluding information about spatial relationships could impact researchers’ findings, since evidence indicates that poor neighbourhood surrounded by other poor neighbourhoods affect people differently than do poor neighbourhoods surrounded by less disadvantaged areas.

Roquette *et al*^10^ conducted a systematised review of 180 studies on cancer spatial epidemiology, analysing geographical data aggregation, risk factors and spatial analysis methods. Most studies used aggregated geographical data, with demographic, socioeconomic and environmental factors being the most frequently examined risk factors, while physiological and genetic factors were analysed more than individual behaviours. Studies usually explored cancer morbidity/mortality associations with risk factors, though findings remained inconclusive. Bayesian models (BYM) and Kriging were commonly used for smoothed rate estimation, while spatial scan statistics and Moran’s I were mostly used in cluster analysis. Various statistical methods were applied in risk factor and environmental analysis, including hierarchical modelling, multilevel modelling, logistic regression and geographically weighted regression. The review highlighted the lack of consensus on cancer risk factors and approaches, influenced by study objectives and data availability.

Zahnd and McLafferty^5^ conducted a systematic review of 122 US-focused cancer outcome studies (2002–2016) to assess how contextual factors are considered, analysing geographical scale, contextual variables and quantification methods. Census tracts were the most commonly used geographical scale, while multilevel models frequently examined socioeconomic factors, healthcare access, racial/ethnic disparities and rural–urban differences. The authors noted that studies incorporating segregation measures often overlooked spatial effects, potentially biasing results due to spatial autocorrelation. They highlighted the need for improved modelling approaches that account for spatial relationships and recommended advanced multilevel statistical and spatial methods, such as conditional and spatial autoregressive modelling, to better capture diverse contextual influences on cancer outcomes.

Thompson *et al*^79^ conducted a systematic review of 38 US breast cancer epidemiology studies (1994–2017) to assess the use of geospatial methods, analysing research questions, populations, analytical techniques and their strengths and limitations. Census tracts and counties were the most common geographical units, and spatial methods were grouped into four categories: spatial interpolation, global and local spatial statistics, proximity analysis for healthcare access and spatiotemporal analysis. Spatial cluster analysis was the most frequently used method, often followed by non-spatial modelling, such as logistic regression applied to cluster outputs. The more sophisticated methods have demonstrated the ability to identify clusters of cancer outcomes as well as to model the local association of outcomes on possible predictors, while accounting for the spatial autocorrelation. However, few of the existing studies assessed by this systematic review draw on these more advanced methods. The authors highlighted that spatial analysis can enhance the visualisation of high-risk areas and pinpoint heterogeneous risk factors, but despite the availability of sophisticated spatial methods, their integration into breast cancer research remains limited, and conventional non-spatial statistical methods are still the most used.

El Khoury^80^ conducted a systematic review of 25 US studies on geospatial disparities in prostate cancer, analysing Geographic Information Systems (GIS) applications in incidence, mortality and survival research while identifying methods used, gaps and limitations. The primary use of GIS was to pinpoint high-burden areas for targeted public health interventions, with applications categorised into mapping (24 studies), processing (14) and analysis (16). Common geographical scales included counties, census tracts, zip codes, neighbourhoods and census block groups, frequently applying methods like geocoding, binomial kriging and spatial empirical Bayesian smoothing. Spatial analysis methods included cluster identification techniques (Spatial Scan Statistics, Getis-Ord-Gi, local Moran’s I) and geographically weighted regression for spatial variations in risk factors. The review highlighted methodological inconsistencies, particularly in geocoding quality and spatial analysis (eg, SaTScan). Therefore, standardised geocoding approaches were recommended to improve comparability. The review also called for integrating comprehensive databases, incorporating temporal dimensions and combining multiple geospatial methodologies, such as Local Indicator of Spatial Autocorrelation and hierarchical Bayesian spatial modelling.

Bizuayehu *et al*^81^ conducted a scoping review on the spatial analysis of cancer survival, examining geographical variations. They assessed the methods and visualisation techniques applied. The review included 32 studies, focusing on small geographical areas using spatial regression or mapping. Census tracts and counties were the most common geographical units, with 78% of studies analysing a single cancer type—most frequently colorectal, breast, ovarian and prostate cancers. Around 70% of studies were conducted in the Americas, with 90% from high-income countries, predominantly the USA (22 studies), Australia (5), Canada (1) and the UK (1). Spatial survival modelling was used in 25 studies, primarily employing Bayesian spatial survival models and Cox proportional hazards additive models. The review highlighted a growing use of spatial survival methods but noted their limited application to a few countries with well-established cancer registries. Challenges in interpreting spatial analysis results and integrating them into decision-making were identified, particularly in China, Bangladesh and the UK, emphasising the need for expanded training and collaboration among researchers and policymakers.

It is important to remind that cancer maps can facilitate the exploration of environmental determinants associated with cancers and their potential aetiological factors.^82 83^ Over the last decade, the availability of epidemiological and environmental data has, in general, significantly increased, largely due to improvements in data collection and sharing practices across institutions (research centres, governments and private companies). Alongside this growth in data availability, rapid advancements in computer processing power and software capabilities have enhanced researchers’ ability to manage and analyse complex and/or large data sets. The development of open-source GIS software and statistical spatial algorithms has enabled spatial and statistical analysis, along with advanced data visualisation. These tools were mostly advanced in the last 15–20 years,^7 82^ and earlier studies may have suffered due to a lack of availability of such data and spatial tools. As mentioned previously, there are still challenges in the use and integration of spatial approaches into policy and decision-making.

One of the most advanced statistical methods that were applied to the geographical analyses of cancers in the UK was joint disease mapping. We have identified two studies^11 55^ covering the Yorkshire region of England. In the joint disease mapping approach, inference is strengthened by borrowing information between related disease and health outcomes,^55^ as it considers spatial correlation of disease rates among neighbouring areas, improving the model with more precise parameter estimates.^11 84^ Joint models allow for simultaneously addressing multiple types of cancers within the same study framework. For example, Chamanpara *et al*^84^ analysed the joint spatial variation of incidence rates for oesophagus and gastric cancer. They revealed geographical differences in cancers, with the northern half of the province at a higher risk than the southern half. This approach highlighted areas with high incidence rates, enhancing understanding of risk factors and revealing nuanced patterns in risk distributions, accounting for shared and specific factors, such as demographic and behavioural factors.

In another study, Mahaki *et al*^85^ explored the spatial and temporal patterns of seven common cancers in Iran from 2005 to 2009. The model improved when including spatial parameters controlling for the interactions between cancers.

The North West presents an elevated incidence for certain types of cancer, particularly for oesophagus, liver, stomach, bladder, lung, trachea and bronchus cancers.^12^ The studies conducted in this region and included in this review do not encompass the variety of cancers existent there, such as head and neck cancer, cervix uteri, colorectal and melanoma of skin.^12^ Blood and lymphoid cell cancers were of major interest not only in the North West, but for the other included studies conducted in the UK.^2728 30^,^35 37^ Among the reasons for this interest was to investigate the potential association with environmental factors, like the exposure of proximity to nuclear installations, magnetic fields or industries, or to investigate claims of possible clusters including population density and the role of infectious agents.^43^ Studies assessing lung,^11 17 35 40 46 55 60 69 74^ liver,^41 54 70^ stomach^11 40 42 46^ and bladder^11 39 40^ were usually conducted across a broader scale, encompassing the nine regions of England, or cohorts for the entire UK.

Regarding the geographical level factors generally considered as potential risk factors for cancer outcomes, they were similar in our included studies, with demographic, environmental and socioeconomic factors being the major factors identified. Lifestyle and behaviour were less explored in the included studies, likely due to the limited availability of such information on a fine-level scale or individual level. On the other hand, demographic, environmental and socioeconomic factors are easily accessible at the population level, allowing more studies with a geographical approach to include them in their analyses. For the studies focusing on the North West, the demographic factors used were usually age and sex, and socioeconomic factors used were related to unemployment, income deprivation and percentage of households in poverty. Population density and proximity to nuclear installations were identified as potential environmental risk factors, especially regarding the presence of two nuclear installations, Sellafield (Cumbria) and Heysham (Lancashire). For the remaining studies conducted in other regions or at different scales, demographic, socioeconomic and environmental factors encompassed a broader variety of factors, including ethnicity,^70^ exposure to sunshine and radon,^49^ proximity to radio and television transmitters^40^ or proximity to heavy industries.^60^

## Conclusion

Most of the 52 articles included in this systematic mapping review considered only England as the study area and focused on a single cancer system only. Few studies employed spatially explicit methods, which account for the spatial arrangement, relationships and interactions between cancer incidence and their determinants. Further, we noticed that most studies did not account for the co-occurrence of cancers. While the North West region presents a high incidence of oesophagus, liver, stomach, bladder, lung, trachea and bronchus cancers, the other cancers were not the subject of areal geographical analyses. In most studies analysed, the use of explicit spatial approaches was lacking, without a clear explanation provided for their absence. As demonstrated in this mapping review, this issue should be considered in future research studies and statistical analyses in the UK. These methods can contribute towards greater understanding of the spatial patterns of cancer distribution, providing insights into potential risk factors and informing preventative strategies, interventions and the allocation of resources to target areas.

## Supplementary material

10.1136/bmjopen-2024-098379 online supplemental file 1

## Data Availability

All data relevant to the study are included in the article or uploaded as supplementary information.
