# Supplementary material for: Systematic mapping review of statistical methods applied to the relationships between cancer diagnosis and geographical level factors in UK
Source: BMJ Open. 2025 Jul 6;15(7):e098379. doi: 10.1136/bmjopen-2024-098379 (PMC12230968; doi:10.1136/bmjopen-2024-098379)
Supplement: online supplemental file 1 [file bmjopen-15-7-s001.docx]

# SUPPLEMENTARY FILE 1

INCLUSION AND EXCLUSION CRITERIA FOR THE SCREENING PROCESS

| Inclusion criteria | (i) focused on the constituent countries of the UK (England, Wales, Scotland and Northern Ireland) and its major regions (e.g. the North West); (ii) compared cancer(s) outcomes with demographic, environmental, behavioural and socioeconomic characteristics by applying methods to identify their spatial association; (iii) reported cancer prevalence, incidence rates, relative risk or odds ratios for a risk factor or to an average level of cancer. |
| --- | --- |
| Exclusion criteria | (i) studies were excluded if they were non-UK or if they included the UK as part of a comparison to other countries; (ii) focused on methods or technologies for cancer detection/diagnosis and screening, exams or treatment/interventions (including palliative care); (iii) assessed risk regarding biological, genetic, infection or comorbidity factors; (iv) assessed clinical outcomes, management/experience of care, cost-effectiveness of medication or treatments; (v) assessed the impact of awareness and campaigns; (vi) evaluated systems or databases and focused on comparing/evaluating different approaches; (vii) considered the relationship between cancer and geographic factors without a spatial distribution/variation or mapping approach; (viii) assessed psychological effects; (ix) reported a descriptive analysis of trends only; (x) considered cancer in animals; (xi) addressed broader health outcomes than solely cancer. |

# SUPPLEMENTARY FILE 2

SEARCH STRATEGY SUGGESTED IN THE PROTOCOL

(MH "Neoplasms+/DI/EP") OR TI ( ( ((HNC OR cancer* OR carcino* OR onco* OR tumor* OR tumour* OR malignan* OR oncol* OR metastasis OR myeloma* OR neoplas* OR tumefaction ) N5 (diagnos* OR identif* OR confirm* OR develop*)) ) ) OR AB ( ( ((HNC OR cancer* OR carcino* OR onco* OR tumor* OR tumour* OR malignan* OR oncol* OR metastasis OR myeloma* OR neoplas* OR tumefaction ) N5 (diagnos* OR identif* OR confirm* OR develop*)) ) )

AND

( (MH "Observational Studies as Topic") OR (MH "Case-Control Studies+") OR (MH "Cross-Sectional Studies") OR (MH "Cohort Studies+") ) OR TI (( "observational" OR "Case-control" OR "Cross-sectional" OR "crosssectional" OR "cross sectional" OR "cohort" OR "ecological") N2 stud*) OR "rapid review" OR "systematic review" OR "cancer registr*") OR AB (( "observational" OR "Case-control" OR "Cross-sectional" OR "crossectional" OR "cross sectional" OR "cohort" OR "ecological") N2 stud*) OR "rapid review" OR "systematic review" OR "cancer registr*")

AND

(MH "United Kingdom+") OR TI ( ( "united kingdom" OR UK OR england OR Britain OR wales OR "northern ireland" OR scotland ) ) OR AB ( ( "united kingdom" OR UK OR england OR Britain OR wales OR "northern ireland" OR scotland ) )

AND

( ( (MH "Healthcare Disparities") OR (MH "Health Status+") OR (MH "Demography+") OR (MH "Socioeconomic Factors+") OR (MH "Sociodemographic Factors") OR (MH "Social Vulnerability") OR (MH "Neighborhood Characteristics") OR (MH "Ethnic and Racial Minorities") OR (MH "Cultural Deprivation") OR (MH "Food Assistance") OR (MH "Social Status") OR (MH "Social Segregation+") OR (MH "Poverty+") OR (MH "Crime+") OR (MH "Quality of Life") OR (MH "Social Control Policies+") OR (MH "Smoke-Free Policy") OR (MH "Sex Distribution+") OR (MH "Health Status+") OR (MH "Health Inequities+") OR (MH "Censuses") OR (MH "Age Distribution") OR (MH "Population+") OR (MH "Health+") OR (MH "Ethnicity") OR (MH "Population Groups") OR (MH "Vital Statistics+") OR (MH "Risk Assessment+") OR (MH "Urban Health") OR (MH "Suburban Health") OR (MH "Rural Health") OR (MH "Minority Health") OR (MH "Veterans Health") OR (MH "Social Determinants of Health") OR (MH "Public Health") OR (MH "Oral Health") OR (MH "One Health") OR (MH "Occupational Health") OR (MH "Military Health") OR (MH "Men's Health") OR (MH "Infant Health") OR (MH "Holistic Health") OR (MH "Global Health") OR (MH "Family Health") OR (MH "Child Health") OR (MH "Adolescent Health") OR (MH "Risk Evaluation and Mitigation") OR (MH "Healthcare Failure Mode and Effect Analysis") OR (MH "Adverse Outcome Pathways") ) ) OR TI ( ( "Built Environment" OR "Macroenvironment" OR "Macro Environment" OR "Macro-Environment" OR Demograph* OR Population* OR Socio* OR Econom* OR Environment* OR Geographic* OR behaviour* OR disparit* OR deprivation OR poverty ) ) OR AB ( ("Built Environment" OR "Macroenvironment" OR "Macro Environment" OR "Macro-Environment" OR Demograph* OR Population* OR Socio* OR Econom* OR Environment* OR Geographic* OR behaviour* OR disparit* OR deprivation OR poverty ) )

Search adapted to MEDLINE

S1 (MH"Neoplasms+/DI/EP") OR TI ( ( (HNC OR cancer* OR carcino* OR onco* OR tumor* OR tumour* OR malignan* OR oncol* OR metastasis OR myeloma* OR neoplas* OR tumefaction ) N5 (diagnos* OR identif* OR confirm* OR develop*) ) ) OR AB ( ( (HNC OR cancer* OR carcino* OR onco* OR tumor* OR tumour* OR malignan* OR oncol* OR metastasis OR myeloma* OR neoplas* OR tumefaction ) N5 (diagnos* OR identif* OR confirm* OR develop*) ) )

S2 ( ( (MH "Observational Studies as Topic") OR (MH "Case-Control Studies+") OR (MH "Cross-Sectional Studies") OR (MH "Cohort Studies+") ) ) OR TI ( ( ( (( "observational" OR "Case-control" OR "Cross-sectional" OR "crosssectional" OR "cross sectional" OR "cohort" OR "ecological") N2 stud*) OR "rapid review" OR "systematic review" OR "cancer registr*") ) ) ) ) OR AB ( ( ( (( "observational" OR "Case-control" OR "Cross-sectional" OR "crosssectional" OR "cross sectional" OR "cohort" OR "ecological") N2 stud*) OR "rapid review" OR "systematic review" OR "cancer registr*") ) ) ) )

S3 (MH "United Kingdom+") OR TI ( ( ("united kingdom" OR UK OR england OR Britain OR wales OR "northern ireland" OR scotland) ) ) OR AB ( ( ("united kingdom" OR UK OR england OR Britain OR wales OR "northern ireland" OR scotland) ) )

S4 ( ( (MH "Healthcare Disparities") OR (MH "Health Status+") OR (MH "Demography+") OR (MH "Socioeconomic Factors+") OR (MH "Sociodemographic Factors") OR (MH "Social Vulnerability") OR (MH "Neighborhood Characteristics") OR (MH "Ethnic and Racial Minorities") OR (MH "Cultural Deprivation") OR (MH "Food Assistance") OR (MH "Social Status") OR (MH "Social Segregation+") OR (MH "Poverty+") OR (MH "Crime+") OR (MH "Quality of Life") OR (MH "Social Control Policies+") OR (MH "Smoke-Free Policy") OR (MH "Sex Distribution+") OR (MH "Health Status+") OR (MH "Health Inequities+") OR (MH "Censuses") OR (MH "Age Distribution") OR (MH "Population+") OR (MH "Health+") OR (MH "Ethnicity") OR (MH "Population Groups") OR (MH "Vital Statistics+") OR (MH "Risk Assessment+") OR (MH "Urban Health") OR (MH "Suburban Health") OR (MH "Rural Health") OR (MH "Minority Health") OR (MH "Veterans Health") OR (MH "Social Determinants of Health") OR (MH "Public Health") OR (MH "Oral Health") OR (MH "One Health") OR (MH "Occupational Health") OR (MH "Military Health") OR (MH "Men's Health") OR (MH "Infant Health") OR (MH "Holistic Health") OR (MH "Global Health") OR (MH "Family Health") OR (MH "Child Health") OR (MH "Adolescent Health") OR (MH "Risk Evaluation and Mitigation") OR (MH "Healthcare Failure Mode and Effect Analysis") OR (MH "Adverse Outcome Pathways") ) ) OR TI ( ( ( "Built Environment" OR "Macroenvironment" OR "Macro Environment" OR "Macro-Environment" OR Demograph* OR Population* OR Socio* OR Econom* OR Environment* OR Geographic* OR behaviour* OR disparit* OR deprivation OR poverty ) ) ) OR AB ( ( ( "Built Environment" OR "Macroenvironment" OR "Macro Environment" OR "Macro-Environment" OR Demograph* OR Population* OR Socio* OR Econom* OR Environment* OR Geographic* OR behaviour* OR disparit* OR deprivation OR poverty ) ) )

S5 S1 AND S2 AND S3 AND S4

Search adapted to Web of Science/SSCI

1: (TI=(HNC OR cancer* OR carcino* OR onco* OR tumor* OR tumour* OR malignan* OR oncol* OR metastasis OR myeloma* OR neoplas* OR tumefaction NEAR diagnos* OR identif* OR confirm* OR develop*)) OR AB=(HNC OR cancer* OR carcino* OR onco* OR tumor* OR tumour* OR malignan* OR oncol* OR metastasis OR myeloma* OR neoplas* OR tumefaction NEAR diagnos* OR identif* OR confirm* OR develop*)

2: (TI=("observational" OR "Case-control" OR "Cross-sectional" OR "crosssectional" OR "cross sectional" OR "cohort" OR "ecological" NEAR stud* OR "rapid review" OR "systematic review" OR "cancer registr*")) OR AB=("observational" OR "Case-control" OR "Cross-sectional" OR "crosssectional" OR "cross sectional" OR "cohort" OR "ecological" NEAR stud* OR "rapid review" OR "systematic review" OR "cancer registr*")

3: (TI=("united kingdom" OR UK OR england OR Britain OR wales OR "northern ireland" OR scotland)) OR AB=("united kingdom" OR UK OR england OR Britain OR wales OR "northern ireland" OR scotland)

4: (TI=("Built Environment" OR "Macroenvironment" OR "Macro Environment" OR "Macro-Environment" OR Demograph* OR Population* OR Socio* OR Econom* OR Environment* OR Geographic* OR behaviour* OR disparit* OR deprivation OR poverty)) OR AB=("Built Environment" OR "Macroenvironment" OR "Macro Environment" OR "Macro-Environment" OR Demograph* OR Population* OR Socio* OR Econom* OR Environment* OR Geographic* OR behaviour* OR disparit* OR deprivation OR poverty)

5: #1 AND #2 AND #3 AND #4

Search adapted to SocINDEX

S1: (MH "Neoplasms+/DI/EP") OR TI ( ( ( ((HNC OR cancer* OR carcino* OR onco* OR tumor* OR tumour* OR malignan* OR oncol* OR metastasis OR myeloma* OR neoplas* OR tumefaction ) N5 (diagnos* OR identif* OR confirm* OR develop*)) ) ) ) OR AB ( ( ( ((HNC OR cancer* OR carcino* OR onco* OR tumor* OR tumour* OR malignan* OR oncol* OR metastasis OR myeloma* OR neoplas* OR tumefaction ) N5 (diagnos* OR identif* OR confirm* OR develop*)) ) ) )

S2: ( ( (MH "Observational Studies as Topic") OR (MH "Case-Control Studies+") OR (MH "Cross-Sectional Studies") OR (MH "Cohort Studies+") ) ) OR TI ( (( "observational" OR "Case-control" OR "Cross-sectional" OR "crosssectional" OR "cross sectional" OR "cohort" OR "ecological") N2 stud*) OR "rapid review" OR "systematic review" OR "cancer registr*") ) OR AB ( (( "observational" OR "Case-control" OR "Cross-sectional" OR "crossectional" OR "cross sectional" OR "cohort" OR "ecological") N2 stud*) OR "rapid review" OR "systematic review" OR "cancer registr*") )

S3: (MH "United Kingdom+") OR TI ( ( ( "united kingdom" OR UK OR england OR Britain OR wales OR "northern ireland" OR scotland ) ) ) OR AB ( ( ( "united kingdom" OR UK OR england OR Britain OR wales OR "northern ireland" OR scotland ) ) )

S4: ( ( ( (MH "Healthcare Disparities") OR (MH "Health Status+") OR (MH "Demography+") OR (MH "Socioeconomic Factors+") OR (MH "Sociodemographic Factors") OR (MH "Social Vulnerability") OR (MH "Neighborhood Characteristics") OR (MH "Ethnic and Racial Minorities") OR (MH "Cultural Deprivation") OR (MH "Food Assistance") OR (MH "Social Status") OR (MH "Social Segregation+") OR (MH "Poverty+") OR (MH "Crime+") OR (MH "Quality of Life") OR (MH "Social Control Policies+") OR (MH "Smoke-Free Policy") OR (MH "Sex Distribution+") OR (MH "Health Status+") OR (MH "Health Inequities+") OR (MH "Censuses") OR (MH "Age Distribution") OR (MH "Population+") OR (MH "Health+") OR (MH "Ethnicity") OR (MH "Population Groups") OR (MH "Vital Statistics+") OR (MH "Risk Assessment+") OR (MH "Urban Health") OR (MH "Suburban Health") OR (MH "Rural Health") OR (MH "Minority Health") OR (MH "Veterans Health") OR (MH "Social Determinants of Health") OR (MH "Public Health") OR (MH "Oral Health") OR (MH "One Health") OR (MH "Occupational Health") OR (MH "Military Health") OR (MH "Men's Health") OR (MH "Infant Health") OR (MH "Holistic Health") OR (MH "Global Health") OR (MH "Family Health") OR (MH "Child Health") OR (MH "Adolescent Health") OR (MH "Risk Evaluation and Mitigation") OR (MH "Healthcare Failure Mode and Effect Analysis") OR (MH "Adverse Outcome Pathways") ) ) ) OR TI ( ( ( "Built Environment" OR "Macroenvironment" OR "Macro Environment" OR "Macro-Environment" OR Demograph* OR Population* OR Socio* OR Econom* OR Environment* OR Geographic* OR behaviour* OR disparit* OR deprivation OR poverty ) ) ) OR AB ( ( ("Built Environment" OR "Macroenvironment" OR "Macro Environment" OR "Macro-Environment" OR Demograph* OR Population* OR Socio* OR Econom* OR Environment* OR Geographic* OR behaviour* OR disparit* OR deprivation OR poverty ) ) )

S5: S1 AND S2 AND S3 AND S4

Search adapted to CINAHL

S1: (MH "Neoplasms+/DI/EP") OR TI ( ( ( ((HNC OR cancer* OR carcino* OR onco* OR tumor* OR tumour* OR malignan* OR oncol* OR metastasis OR myeloma* OR neoplas* OR tumefaction ) N5 (diagnos* OR identif* OR confirm* OR develop*)) ) ) ) OR AB ( ( ( ((HNC OR cancer* OR carcino* OR onco* OR tumor* OR tumour* OR malignan* OR oncol* OR metastasis OR myeloma* OR neoplas* OR tumefaction ) N5 (diagnos* OR identif* OR confirm* OR develop*)) ) ) )

S2: ( ( (MH "Observational Studies as Topic") OR (MH "Case-Control Studies+") OR (MH "Cross-Sectional Studies") OR (MH "Cohort Studies+") ) ) OR TI ( (( "observational" OR "Case-control" OR "Cross-sectional" OR "crosssectional" OR "cross sectional" OR "cohort" OR "ecological") N2 stud*) OR "rapid review" OR "systematic review" OR "cancer registr*") ) OR AB ( (( "observational" OR "Case-control" OR "Cross-sectional" OR "crossectional" OR "cross sectional" OR "cohort" OR "ecological") N2 stud*) OR "rapid review" OR "systematic review" OR "cancer registr*") )

S3: (MH "United Kingdom+") OR TI ( TI ( ( "united kingdom" OR UK OR england OR Britain OR wales OR "northern ireland" OR scotland ) ) ) OR AB ( ( ( "united kingdom" OR UK OR england OR Britain OR wales OR "northern ireland" OR scotland ) ) )

S4: ( ( ( (MH "Healthcare Disparities") OR (MH "Health Status+") OR (MH "Demography+") OR (MH "Socioeconomic Factors+") OR (MH "Sociodemographic Factors") OR (MH "Social Vulnerability") OR (MH "Neighborhood Characteristics") OR (MH "Ethnic and Racial Minorities") OR (MH "Cultural Deprivation") OR (MH "Food Assistance") OR (MH "Social Status") OR (MH "Social Segregation+") OR (MH "Poverty+") OR (MH "Crime+") OR (MH "Quality of Life") OR (MH "Social Control Policies+") OR (MH "Smoke-Free Policy") OR (MH "Sex Distribution+") OR (MH "Health Status+") OR (MH "Health Inequities+") OR (MH "Censuses") OR (MH "Age Distribution") OR (MH "Population+") OR (MH "Health+") OR (MH "Ethnicity") OR (MH "Population Groups") OR (MH "Vital Statistics+") OR (MH "Risk Assessment+") OR (MH "Urban Health") OR (MH "Suburban Health") OR (MH "Rural Health") OR (MH "Minority Health") OR (MH "Veterans Health") OR (MH "Social Determinants of Health") OR (MH "Public Health") OR (MH "Oral Health") OR (MH "One Health") OR (MH "Occupational Health") OR (MH "Military Health") OR (MH "Men's Health") OR (MH "Infant Health") OR (MH "Holistic Health") OR (MH "Global Health") OR (MH "Family Health") OR (MH "Child Health") OR (MH "Adolescent Health") OR (MH "Risk Evaluation and Mitigation") OR (MH "Healthcare Failure Mode and Effect Analysis") OR (MH "Adverse Outcome Pathways") ) ) ) OR TI ( ( ( "Built Environment" OR "Macroenvironment" OR "Macro Environment" OR "Macro-Environment" OR Demograph* OR Population* OR Socio* OR Econom* OR Environment* OR Geographic* OR behaviour* OR disparit* OR deprivation OR poverty ) ) ) OR AB ( ( ("Built Environment" OR "Macroenvironment" OR "Macro Environment" OR "Macro-Environment" OR Demograph* OR Population* OR Socio* OR Econom* OR Environment* OR Geographic* OR behaviour* OR disparit* OR deprivation OR poverty ) ) )

S5: S1 AND S2 AND S3 AND S4

# SUPPLEMENTARY FILE 3

CORE DATA EXTRACTED

| **Concept (Data Extracted)** | **Definition** |
| --- | --- |
| Year | Bibliographic details |
| Title | Bibliographic details |
| Journal | Bibliographic details |
| Vol./Issue | Bibliographic details |
| Pags | Bibliographic details |
| DOI | Bibliographic details |
| Key words | Key words defined by the authors |
| Type | Type of publication (e.g.: article, short report) |
| Aim | Aim/objective of the study |
| Study design | Study design (e.g.: ecological, case-control, cohort) |
| Where - Study area | Geographical region or location where the research or analysis was conducted |
| Data source | Origin/institution from which the data were collected or provided by (e.g.: National Cancer Registration and Analysis Service database (NCRAS); Manchester Children's Tumour Registry) |
| Aggregation level | Geographical unit to which the data were aggregated for analysis (e.g.: Regions; Local Authorities; Wards) |
| Population | Population analysed (population with cancer diagnosis) |
| Years/period considered in the study | Period considered for analysis |
| Cancer Types | Cancer types, aggregated into categories (e.g.: blood and Lymphoid cells cancers; skin cancers; liver cancers) |
| Health Data measurement | Data obtained from the source (e.g.: cases; incidence rates) |
| Geographical level factors | Aggregated into categories (e.g.: Socioeconomic factors, demographic factors, environmental factors). These factors might be (or not) confirmed as a risk factor. |
| Methods applied | Methods used to evaluate the cancer’s spatial distribution, variation or interaction with socioeconomic, demographic, environmental and behavioural factors (e.g.: regression analysis; cluster detection; correlation analysis) |
| Potential risk factors | Might be the same as one or more geographical level factors analysed, if detected significant association with cancer (e.g.: socioeconomic factors, environmental factors) |
| Outcomes | Result of the analysis (e.g.: incidence rates; relative risk; odds ratio) |
| Relevant Figures | Figures presenting the results of the cancer’s relationship with geographical level factors (e.g.: plots or maps), indicating their number in the original study. |
| Relevant Tables | Result of the analysis (e.g.: tables presenting results of regressions), indicating their number in the original study. |
| Presentation of main results | Form of presenting the results of the cancer’s relationship with geographical level factors (e.g.: tables, plots and/or maps) |
| Main conclusions/findings | Paragraph synthesizing the main findings or stating the conclusions |
| With what other articles does it strongly relate? | Indicator of whether the authors mention a clear relation to a previous study that might have inspired their research (e.g.: updating or expanding a previous research) |
| Research innovation | Indicator of whether the authors clearly mention innovation in their study |

# SUPPLEMENTARY FILE 4

NUMERICAL DATA EXTRACTED – SPATIALLY EXPLICIT APPROACHES

| **Author** | **Population** | **Cancer type** | **Age** | **Study site** | **Period studied** | **Adjusted covariates** | **Spatial explicit method** | **Model comparison** | **Notes** |  |
| --- | --- | --- | --- | --- | --- | --- | --- | --- | --- | --- |
| **Sehmer et al. 2014** | 435 patients | Glioma | 18–89 years old | Lancashire and South Cumbria | 2006-2010 | Age | Spatio-temporal cluster detection (SatScan) | Not applicable | None |  |
|  | **OUTCOME MEASUREMENT** | | | | | | | | |  |
|  | **Age-standardized incidence rates** | | | | | | | | |  |
|  | 7.10 per 100,000 (95% CI, 6.47–7.80). | | | | | | | | |  |
| **Author** | **Population** | **Cancer type** | **Age** | **Study site** | **Period studied** | **Adjusted covariates** | **Spatial explicit method** | **Model comparison** | **Notes** |  |
| **Toledano et al. 2001** | 662 cases | Testicular cancer | 20–49 years old | Great Britain | 1974 | Age and deprivation | Bayesian hierarchical modelling | DIC (Computed only for two specific models in North West Thames and Yorkshire cancer registries due to computational limitations) | None |  |
|  |  |  |  |  |  |  |  |  |  |  |
|  |  |  |  |  |  |  |  |  |  |  |
|  | 1290 cases |  |  |  | 1991 |  |  |  |  |  |
|  |  |  |  |  |  |  |  |  |  |  |
|  |  |  |  |  |  |  |  |  |  |  |
|  | **OUTCOME MEASUREMENT** | | | | | | | | |  |
|  | **Spatial variation in relative risks (RR) of testicular cancer adjusted for age, relative deprivation (using Carstairs’ scores) and** | | | | | | | | |  |
|  | **cancer registry across 10,530 electoral wards in Great Britain (1975–1991), using hierarchical Bayesian methods for the smoothed estimates** | | | | | | | | |  |
|  |  | | | | **Observed** | | **Expected** | **Unsmoothed RR** | **Smoothed RR** |  |
|  | **Minimum** | | | | 0 | | 0.01 | 0 | 0.79 |  |
|  | **5th percentile** | | | | 0 | | 0.59 | 0 | 0.93 |  |
|  | **Median** | | | | 1 | | 1.15 | 0.78 | 0.99 |  |
|  | **95th percentile** | | | | 2 | | 1.98 | 1.53 | 1.1 |  |
|  | **Maximum** | | | | 35 | | 9.14 | 29.2 | 2.81 |  |
|  | **Mean** | | | | 1.45 | | 1.45 | 1.01 | 1 |  |

NUMERICAL DATA EXTRACTED – SPATIALLY EXPLICIT APPROACHES (CONT.)

| **Author** | **Population** | **Cancer type** | **Age** | **Study site** | **Period studied** | **Adjusted covariates** | **Spatial explicit method** | **Model comparison** | **Notes** | | |
| --- | --- | --- | --- | --- | --- | --- | --- | --- | --- | --- | --- |
| **Richardson, Abellan & Best 2006** | 626 wards are considered | Lung cancer | Not mentioned (see Notes) | Yorkshire | 1981-1999 | Age and sex | Bayesian hierarchical modelling | DIC. The models differ in their assumption of the space–time structure and the inclusion or not of a heterogeneity term. | **Population:** the average number of observed cases per ward per year is 4.1 for males (range 0–32) and 1.9 for females (0–17). 626 wards are considered in the analysis | | |
|  |  |  |  |  |  |  |  |  | **Ages:** All ages might be considered as the authors calculated the expected counts based on average age–sex-specific. | | |
|  |  |  |  |  |  |  |  |  | **Model comparison**: Model 1 (simple additive decomposition for the shared part without heterogeneity): 6100.4; Model 2 ( include space–time interaction terms only on the shared component): 6043.7; Model 1h (adding heterogeneity terms to model 1): 6057.1; Model 2h (adding heterogeneity terms to model 2): 6045.3 | | |
|  | **OUTCOME MEASUREMENT** | | | | | | | | | | |
|  | **RAW AND SMOOTHED SIR** | | | | | | | | | | |
|  |  | |  |  | **Raw SIR** | **Posterior Median Smoothed SIR (Model 2)** |  |  |  | **Raw SIR** | **Posterior Median Smoothed SIR (Model 2)** |
|  | **Gender** | | **Period** | **SIR range** | **Number of wards** | **Number of wards** | **Gender** | **Period** | **SIR range** | **Number of wards** | **Number of wards** |
|  | **Male** | | 1981-1985 | <0.85 | 224 | 198 | **Female** | 1981-1985 | <0.85 | 388 | 476 |
|  |  |  |  | 0.85-0.95 | 59 | 91 |  |  | 0.85-0.95 | 48 | 72 |
|  |  |  |  | 0.95-1.05 | 85 | 95 |  |  | 0.95-1.05 | 61 | 39 |
|  |  |  |  | 1.05-1.17 | 49 | 77 |  |  | 1.05-1.17 | 39 | 19 |
|  |  |  |  | >1.17 | 209 | 165 |  |  | >1.17 | 90 | 20 |
|  |  |  | 1986-1990 | <0.85 | 272 | 275 |  | 1986-1990 | <0.85 | 298 | 340 |
|  |  |  |  | 0.85-0.95 | 69 | 99 |  |  | 0.85-0.95 | 58 | 94 |
|  |  |  |  | 0.95-1.05 | 65 | 76 |  |  | 0.95-1.05 | 67 | 71 |
|  |  |  |  | 1.05-1.17 | 63 | 77 |  |  | 1.05-1.17 | 59 | 63 |
|  |  |  |  | >1.17 | 157 | 99 |  |  | >1.17 | 144 | 58 |
|  |  |  | 1991-1995 | <0.85 | 316 | 350 |  | 1991-1995 | <0.85 | 258 | 238 |
|  |  |  |  | 0.85-0.95 | 65 | 81 |  |  | 0.85-0.95 | 57 | 101 |
|  |  |  |  | 0.95-1.05 | 69 | 72 |  |  | 0.95-1.05 | 59 | 91 |
|  |  |  |  | 1.05-1.17 | 60 | 71 |  |  | 1.05-1.17 | 65 | 73 |
|  |  |  |  | >1.17 | 116 | 52 |  |  | >1.17 | 187 | 123 |
|  |  |  | 1996-1999 | <0.85 | 357 | 424 |  | 1996-1999 | <0.85 | 233 | 168 |
|  |  |  |  | 0.85-0.95 | 72 | 67 |  |  | 0.85-0.95 | 41 | 124 |
|  |  |  |  | 0.95-1.05 | 59 | 76 |  |  | 0.95-1.05 | 47 | 74 |
|  |  |  |  | 1.05-1.17 | 62 | 35 |  |  | 1.05-1.17 | 59 | 97 |
|  |  |  |  | >1.17 | 76 | 24 |  |  | >1.17 | 246 | 163 |

NUMERICAL DATA EXTRACTED – SPATIALLY EXPLICIT APPROACHES (CONT.)

| **Author** | **Population** | **Cancer type** | **Age** | **Study site** | **Period studied** | **Adjusted covariates** | **Spatial explicit method** | **Model comparison** | **Notes** |
| --- | --- | --- | --- | --- | --- | --- | --- | --- | --- |
| **Downing et al. 2008** | 7444 cases | Oesophagus | Not mentioned | Yorkshire | 1983-2003 | Three components were included to represent differences in smoking (for all six sites), bodyweight/ obesity (for oesophagus, pancreas and kidney cancers) and diet/alcohol consumption (for oesophagus and stomach cancers); Smoothed SIR adjusted for socioeconomic background | Bayesian shared-component model (individual BYM models; joint model) | DIC. Individual BYM models: 19083.04; Joint model: 18980.5 | Standardized incidence ratios (SIR) unsmoothed and smoothed (adjusted for socioeconomic background). Results represented in maps, at wards level |
|  | 15045 cases | Stomach |  |  |  |  |  |  |  |
|  | 8522 cases | Pancreas |  |  |  |  |  |  |  |
|  | 54520 cases | Lung |  |  |  |  |  |  |  |
|  | 5918 cases | Kidney |  |  |  |  |  |  |  |
|  | 15072 cases | Bladder |  |  |  |  |  |  |  |
|  | **OUTCOME MEASUREMENT** | | | | | | | | |
|  | **MEAN AND RANGE OF THE STANDARDIZED INCIDENCE RATIOS (SIR) FOR EACH CANCER** | | | | | | | | |
|  |  | | | | **Mean SIR** | | **Minimum SIR** | | **Maximum SIR** |
|  | **Oesophagus** | | | | 1.06 | | 0.00 | | 3.24 |
|  | **Stomach** | | | | 0.94 | | 0.00 | | 2.55 |
|  | **Pancreas** | | | | 1.02 | | 0.00 | | 2.76 |
|  | **Lung** | | | | 0.92 | | 0.07 | | 2.80 |
|  | **Kidney** | | | | 1.00 | | 0.00 | | 2.79 |
|  | **Bladder** | | | | 1.01 | | 0.00 | | 2.58 |
|  |  | | | | | | | | |
|  | **POSTERIOR MEDIAN (95%CI) RELATIVE RISK OF CANCER INCIDENCE BY TOWSEND QUINTILE** | | | | | | | | |
|  |  |  |  | **I (most affluent)** | | **II** | **III** | **IV** | **V (most deprived)** |
|  | **Oesophagus** | | | 1.00 | | 1.11 (0.99-1.24) | 1.16 (1.03-1.31) | 1.16 (1.02-1.32) | 1.07 (0.93-1.23) |
|  | **Stomach** | | | 1.00 | | 1.11 (1.01-1.22) | 1.24 (1.13-1.37) | 1.27 (1.15-1.41) | 1.33 (1.19-1.48) |
|  | **Pancreas** | | | 1.00 | | 1.12 (1.01-1.24) | 1.09 (0.98-1.22) | 1.10 (0.98-1.24) | 1.03 (0.91-1.16) |
|  | **Lung** | | | 1.00 | | 1.12 (1.04-1.20) | 1.31 (1.21-1.41) | 1.47 (1.35-1.59) | 1.63 (1.47-1.78) |
|  | **Kidney** | | | 1.00 | | 1.09 (0.98-1.22) | 0.96 (0.86-1.09) | 0.97 (0.86-1.10) | 0.89 (0.78-1.01) |
|  | **Bladder** | | | 1.00 | | 1.03 (0.94-1.12) | 1.06 (0.96-1.17) | 0.99 (0.89-1.10) | 0.94 (0.84-1.05) |

NUMERICAL DATA EXTRACTED – SPATIALLY EXPLICIT APPROACHES (CONT.)

| **Author** | **Population** | **Cancer type** | **Age** | **Study site** | **Period studied** | **Adjusted covariates** | **Spatial explicit method** | **Model comparison** | **Notes** |
| --- | --- | --- | --- | --- | --- | --- | --- | --- | --- |
| **McNally et al. 2003** | 910 cases | Leukaemias and Lymphomas | 0-14 years old | North-west England | 1976-2000 | Not mentioned | Potthoff–Whittinghill test to identify spatial clustering | Not applicable | None |
|  | **OUTCOME MEASUREMENT** | | | | | | | | |
|  | **LOCALIZED EXCESSES OF CASES** | | | | | | | | |
|  | **Cancer type** | | | **Year** | | **P-value** | **Localized excesses of cases** | | |
|  | **Acute Leukaemias** | | | 1976–1985 | | P=0.78 | No evidence for the general presence of localized excesses/spatial clustering | | |
|  |  |  |  | 1986–2000 | | P=0.37 | No evidence for the general presence of localized excesses/spatial clustering | | |
|  | **Hodgkin's Disease** | | | 1986–2000 | | P=0.04 | Exhibited evidence for the general presence of localized excesses/spatial clustering | | |
|  | **Non-Hodgkin's lymphoma** | | | 1976–1985 | | P=0.67 | No evidence for the general presence of localized excesses/spatial clustering | | |
|  |  |  |  | 1986–2000 | | P=0.56 | No evidence for the general presence of localized excesses/spatial clustering | | |
| **Author** | **Population** | **Cancer type** | **Age** | **Study site** | **Period studied** | **Adjusted covariates** | **Spatial explicit method** | **Model comparison** | **Notes** |
| **Muir et al. 2004** | Not mentioned | Female breast cancer | >45 years old | Lincolnshire and Leicestershire | 1989–1991 | Age | Black–White BW join-count statistic and Moran's I (Spatial autocorrelation) | Not applicable | A negative black–white statistic and a positive Moran I coefficient defines positive spatial autocorrelation |
|  | **OUTCOME MEASUREMENTS** | | | | | | | | |
|  | **RESULTS OF SPATIAL AUTOCORRELATION TESTS FOR BREAST CANCER INCIDENCE IN LINCOLNSHIRE AND LEICESTERSHIRE** | | | | | | | | |
|  | **Counties** | | | **Black-white statistic*** | | **P value B-W** | **Moran I statistic*** | **P value Moran I** | **The county-level SIR (per 1000 women)** |
|  | **Lincolnshire** | | | 1.716 | | 0.96 | 0.003 | 0.43 | 8.25 |
|  | **Leicestershire** | | | -0.09 | | 0.46 | -0.011 | 0.45 | 7.6 |
|  | * A negative black–white statistic and a positive Moran I coefficient defines positive spatial autocorrelation | | | | | | | | |

NUMERICAL DATA EXTRACTED – SPATIALLY EXPLICIT APPROACHES (CONT.)

| **Author** | **Population** | **Cancer type** | **Age** | **Study site** | **Period studied** | **Adjusted covariates** | **Spatial explicit method** | **Model comparison** | **Notes** | | |
| --- | --- | --- | --- | --- | --- | --- | --- | --- | --- | --- | --- |
| **Mangout et al. 1985** | 924 cases | Hodgkin lymphoma | All ages, disaggregated by different age-groups | Greater Manchester | 1962-1976 | Not mentioned | Knox's test for space-time interaction | Not applicable | None | | |
|  | **OUTCOME MEASUREMENT** | | | | | | | | | | |
|  | **SPACE-TIME TABLE FOR 'CLOSE PAIRS' OF CASES DISAGGREGATED BY SEX (OBSERVED AND EXPECTED CUMULATIVE FREQUENCY)** | | | | | | | | | | |
|  |  | | |  | **Distance apart** | | | | | |  |
|  | **Time apart (days)** | | | **Sex** | **< 0.5 km** | | **< 1 km** | | **< 2 km** | | **All pairs** |
|  |  |  |  |  | **O** | **E** | **O** | **E** | **O** | **E** | **Totals** |
|  | **≤ 30** | | | **M** | 5 | 3.2 | 12 | 11.0 | 41 | 37.2 | 1803 |
|  |  |  |  | **F** | **5 | 1.3 | *9 | 4.7 | 17 | 14.7 | 667 |
|  | **≤ 60** | | | **M** | *11 | 6.3 | 29 | 22.0 | 78 | 74.3 | 3597 |
|  |  |  |  | **F** | **7 | 2.6 | 14 | 9.1 | 29 | 28.1 | 1274 |
|  | **≤ 120** | | | **M** | 18 | 12.9 | *56 | 44.6 | 170 | 150.6 | 7291 |
|  |  |  |  | **F** | *10 | 5.2 | 24 | 18.3 | 56 | 56.5 | 2567 |
|  | **≤ 240** | | | **M** | 32 | 25.3 | 93 | 87.7 | 304 | 296.2 | 14340 |
|  |  |  |  | **F** | **20 | 10.2 | *47 | 36.0 | 118 | 111.5 | 5063 |
|  | **≤ 360** | | | **M** | 45 | 37.5 | 142 | 130.2 | 454 | 439.6 | 21287 |
|  |  |  |  | **F** | **25 | 15.0 | 62 | 52.9 | 171 | 163.6 | 7431 |
|  | **≤ 720** | | | **M** | 74 | 72.7 | 248 | 252.3 | 848 | 851.8 | 41242 |
|  |  |  |  | **F** | 37 | 29.2 | 110 | 103.0 | 314 | 318.7 | 14472 |
|  | **All pairs** | | | **M** | 294 |  | 1020 |  | 3444 |  | 166759 |
|  |  |  |  | **F** | 119 |  | 420 |  | 1299 |  | 58996 |
|  | *** Difference significant at the P≤0.05 level** | | | | **M = males** | |  |  |  |  |  |
|  | **** Difference significant at the P≤0.01 level** | | | | **F = females** | |  |  |  |  |  |
|  |  |  |  |  |  |  |  |  |  |  |  |

NUMERICAL DATA EXTRACTED – SPATIALLY EXPLICIT APPROACHES (CONT.)

| **Author** | **Population** | **Cancer type** | **Age** | **Study site** | **Period studied** | **Adjusted covariates** | **Spatial explicit method** | **Model comparison** | **Notes** | | |
| --- | --- | --- | --- | --- | --- | --- | --- | --- | --- | --- | --- |
| **Mangout et al. 1985** | 924 cases | Hodgkin lymphoma | All ages, disaggregated by different age-groups | Greater Manchester | 1962-1976 | Not mentioned | Knox's test for space-time interaction | Not applicable | None | | |
|  | **OUTCOME MEASUREMENT** | | | | | | | | | | |
|  | **SPACE-TIME TABLE FOR 'CLOSE PAIRS' OF CASES DISAGGREGATED BY AGE (OBSERVED AND EXPECTED CUMULATIVE FREQUENCY)** | | | | | | | | | | |
|  |  | | | | **Distance apart** | | | | | |  |
|  | **Time apart (days)** | | | **Sex** | **< 0.5 km** | | **< 1 km** | | **< 2 km** | | **All pairs** |
|  |  |  |  |  | **O** | **E** | **O** | **E** | **O** | **E** | **Totals** |
|  | **≤ 30** | | | **A** | **3 | 0.1 | **3 | 0.1 | **3 | 0.3 | 11 |
|  |  |  |  | **B** | 2 | 1.9 | 5 | 5.9 | 20 | 18.7 | 981 |
|  |  |  |  | **C** | 4 | 2.2 | **15 | 7.7 | 33 | 26.4 | 1192 |
|  | **≤ 60** | | | **A** | **3 | 0.2 | *3 | 0.3 | 3 | 0.7 | 22 |
|  |  |  |  | **B** | 5 | 3.7 | 11 | 11.5 | 34 | 36.4 | 1915 |
|  |  |  |  | **C** | 5 | 4.2 | **24 | 14.8 | 59 | 50.8 | 2296 |
|  | **≤ 120** | | | **A** | **3 | 0.4 | **3 | 0.5 | 3 | 1.2 | 39 |
|  |  |  |  | **B** | 5 | 7.3 | 17 | 22.7 | 62 | 72.2 | 3793 |
|  |  |  |  | **C** | *14 | 8.5 | **43 | 30.1 | 120 | 103.6 | 4679 |
|  | **≤ 240** | | | **A** | **3 | 0.5 | *3 | 0.7 | 3 | 1.9 | 61 |
|  |  |  |  | **B** | 14 | 14.3 | 47 | 44.3 | 132 | 140.6 | 7390 |
|  |  |  |  | **C** | *24 | 17.1 | 70 | 60.3 | 219 | 207.7 | 9381 |
|  | **≤ 360** | | | **A** | **4 | 0.8 | *4 | 1.1 | 5 | 2.8 | 91 |
|  |  |  |  | **B** | 21 | 21.2 | 67 | 65.7 | 204 | 208.6 | 10963 |
|  |  |  |  | **C** | 29 | 25.3 | 73 | 89.3 | 321 | 307.1 | 13875 |
|  | **≤ 720** | | | **A** | **6 | 1.6 | *6 | 2.1 | 8 | 5.6 | 182 |
|  |  |  |  | **B** | 47 | 41.0 | 140 | 127.0 | 392 | 403.0 | 21186 |
|  |  |  |  | **C** | 47 | 49.0 | 190 | 172.7 | 613 | 594.3 | 26850 |
|  | **All pairs** | | | **A** | 7 |  | 9 |  | 24 |  | 780 |
|  |  |  |  | **B** | 168 |  | 520 |  | 1650 |  | 86736 |
|  |  |  |  | **C** | 197 |  | 694 |  | 2388 |  | 107880 |
|  | *** Difference significant at the P≤0.05 level** | | | | **A= aged 0-14 years** | |  |  |  |  |  |
|  | **** Difference significant at the P≤0.01 level** | | | | **B= aged 15-44 years** | |  |  |  |  |  |
|  |  | | | | **C= aged 45 years and over** | |  |  |  |  |  |

# SUPPLEMENTARY FILE 5

QUALITY APPRAISAL ASSESSMENT TOOLS

**NEWCASTLE - OTTAWA QUALITY ASSESSMENT SCALE**

**CASE CONTROL STUDIES**

Note: A study can be awarded a maximum of one star for each numbered item within the Selection and

Exposure categories. A maximum of two stars can be given for Comparability.

**Selection**

1) Is the case definition adequate?

a) yes, with independent validation **¯**

b) yes, eg record linkage or based on self reports

c) no description

2) Representativeness of the cases

a) consecutive or obviously representative series of cases **¯**

b) potential for selection biases or not stated

3) Selection of Controls

a) community controls **¯**

b) hospital controls

c) no description

4) Definition of Controls

a) no history of disease (endpoint) **¯**

b) no description of source

**Comparability**

1) Comparability of cases and controls on the basis of the design or analysis

a) study controls for _______________ (Select the most important factor.) **¯**

b) study controls for any additional factor **¯** (This criteria could be modified to indicate specific

control for a second important factor.)

**Exposure**

1) Ascertainment of exposure

a) secure record (eg surgical records) **¯**

b) structured interview where blind to case/control status **¯**

c) interview not blinded to case/control status

d) written self report or medical record only

e) no description

2) Same method of ascertainment for cases and controls

a) yes **¯**

b) no

3) Non-Response rate

a) same rate for both groups **¯**

b) non respondents described

c) rate different and no designation

JBI Critical Appraisal Checklist for studies reporting prevalence data

Reviewer ______________________________________ Date_______________________________

Author_______________________________________ Year_________ Record Number_________

|  | Yes | No | Unclear | Not applicable |
| --- | --- | --- | --- | --- |
| 1. Was the sample frame appropriate to address the target population? | □ | □ | □ | □ |
| 1. Were study participants sampled in an appropriate way? | □ | □ | □ | □ |
| 1. Was the sample size adequate? | □ | □ | □ | □ |
| 1. Were the study subjects and the setting described in detail? | □ | □ | □ | □ |
| 1. Was the data analysis conducted with sufficient coverage of the identified sample? | □ | □ | □ | □ |
| 1. Were valid methods used for the identification of the condition? | □ | □ | □ | □ |
| 1. Was the condition measured in a standard, reliable way for all participants? | □ | □ | □ | □ |
| 1. Was there appropriate statistical analysis? | □ | □ | □ | □ |
| 1. Was the response rate adequate, and if not, was the low response rate managed appropriately? | □ | □ | □ | □ |

Overall appraisal: Include □ Exclude □ Seek further info □

Comments (Including reason for exclusion)

# SUPPLEMENTARY FILE 6

QUALITY APPRAISAL RESULTS (NEWCASTLE-OTTAWA)

|  | 1 Selection | | | | 2 Comparability | 3 Exposure | | |
| --- | --- | --- | --- | --- | --- | --- | --- | --- |
| Study authors, year | Is the case definition adequate? | Representativeness of the cases | Selection of Controls | Definition of Controls | Comparability of cases and controls on the basis of the design or analysis | Ascertainment of exposure | Same method of ascertainment for cases and controls | Non-Response rate |
| UK Childhood Cancer Study Investigators (2000) | a* | a* | a* | a* | study controls for age in years, sex and UKCCS region ** | a* | b | b |
| Alexander FE, McKinney PA, Moncrieff KC, Cartwright RA. (1992) | b | a* | a* | b | study controls for sex, date-of-birth and health district of birth ** | a* | b | b |
| Bithell JF, Murphy MFG, Stiller CA, Toumpakari E, Vincent T, Wakeford R. (2013) | b | a* | a* | a* | study controls for sex, age at diagnosis in years, residential region of Great Britain at the 1981 census and birth year ** | a* | a* | NA |
| Bunch KJ, Keegan TJ, Swanson J, Vincent TJ, Murphy MFG. (2014) | b | a* | a* | a* | study controls for sex, approximate date of birth and birth registration sub-district ** | a* | a* | NA |
| Draper G, Vincent T, Kroll ME, Swanson J. (2005) | b | a* | a* | b | study controls for sex, approximate date of birth, and birth registration district ** | a* | b | NA |
| Edwards R, Pless-Mulloli T, Howel D, Chadwick T, Bhopal R, Harrison R, et al. (2006) | a* | a* | a* | a* | study controls by 5 year age group ** | a* | b | b |
| Elliott P, Shaddick G, Douglass M, de Hoogh K, Briggs DJ, Toledano MB. (2013) | b | a* | a* | a* | study controls frequency-matched on year and region ** | a* | a* | NA |
| Youngson JH, Clayden AD, Myers A, Cartwright RA. (1991) | b | a* | B | a* | study controls for age, sex, year of diagnosis and health district of residence ** | d | a* | NA |

QUALITY APPRAISAL RESULTS (JBI)

|  | Q1 | | | | Q2 | | | | Q3 | | | | Q4 | | | | Q5 | | | | Q6 | | | | Q7 | | | | Q8 | | | | Q9 | | | |
| --- | --- | --- | --- | --- | --- | --- | --- | --- | --- | --- | --- | --- | --- | --- | --- | --- | --- | --- | --- | --- | --- | --- | --- | --- | --- | --- | --- | --- | --- | --- | --- | --- | --- | --- | --- | --- |
| Study authors, year | Y | N | U | NA | Y | N | U | NA | Y | N | U | NA | Y | N | U | NA | Y | N | U | NA | Y | N | U | NA | Y | N | U | NA | Y | N | U | NA | Y | N | U | NA |
| Abdulrahman GOJ. (2014) | X |  |  |  |  |  |  | X | X |  |  |  | X |  |  |  | X |  |  |  | X |  |  |  |  |  |  | X | X |  |  |  |  |  |  | X |
| Alexander FE, Cartwright RA, McKinney PA, Ricketts TJ. (1990) | X |  |  |  |  |  |  | X | X |  |  |  | X |  |  |  | X |  |  |  | X |  |  |  |  |  |  | X | X |  |  |  |  |  |  | X |
| Badrinath P, Day NE, Stockton D. (1999) | X |  |  |  |  |  |  | X | X |  |  |  | X |  |  |  | X |  |  |  | X |  |  |  |  |  |  | X | X |  |  |  |  |  |  | X |
| Bhopal RS, Phillimore P, Moffatt S, Foy C. (1994) | X |  |  |  | X |  |  |  | X |  |  |  | X |  |  |  | X |  |  |  |  |  | X |  |  |  |  | X |  |  | X |  | X |  |  |  |
| Brodbelt A, Greenberg D, Winters T, Williams M, Vernon S, Collins VP. (2015) | X |  |  |  |  |  |  | X | X |  |  |  | X |  |  |  | X |  |  |  | X |  |  |  |  |  |  | X | X |  |  |  |  |  |  | X |
| Bunch KJ, Vincent TJ, Black RJ, Pearce MS, McNally RJQ, McKinney PA, et al. (2014) | X |  |  |  |  |  |  | X | X |  |  |  | X |  |  |  | X |  |  |  | X |  |  |  |  |  |  | X |  |  | X |  |  |  |  | X |
| Burton A, Balach, rakumar VK, Driver RJ, Tataru D, Paley L, et al. (2022) | X |  |  |  |  |  |  | X | X |  |  |  | X |  |  |  | X |  |  |  | X |  |  |  |  |  |  | X | X |  |  |  |  |  |  | X |
| Cartwright RA, Gilman EA, Nicholson P, Allon D. (1999) | X |  |  |  |  |  |  | X | X |  |  |  | X |  |  |  | X |  |  |  | X |  |  |  |  |  |  | X | X |  |  |  |  |  |  | X |
| Chambers AC, Dixon SW, White P, Williams AC, Thomas MG, Messenger DE. (2020) | X |  |  |  |  |  |  | X | X |  |  |  | X |  |  |  | X |  |  |  | X |  |  |  |  |  |  | X | X |  |  |  |  |  |  | X |
| Conway DI, Stockton DL, Warnakulasuriya KAAS, Ogden G, Macpherson LMD (2006) | X |  |  |  |  |  |  | X | X |  |  |  | X |  |  |  | X |  |  |  | X |  |  |  |  |  |  | X | X |  |  |  |  |  |  | X |
| Craft AW, Parker L, Openshaw S, Charlton M, Newell J, Birch JM, et al. (1993) | X |  |  |  |  |  |  | X | X |  |  |  | X |  |  |  | X |  |  |  | X |  |  |  |  |  |  | X | X |  |  |  |  |  |  | X |
| Dolk H, Elliott P, Shaddick G, Walls P, Thakrar B. (1997) | X |  |  |  |  |  |  | X | X |  |  |  | X |  |  |  | X |  |  |  | X |  |  |  |  |  |  | X | X |  |  |  |  |  |  | X |
| Dolk H, Shaddick G, Walls P, Grundy C, Thakrar B, Kleinschmidt I, et al. (1997) | X |  |  |  |  |  |  | X | X |  |  |  | X |  |  |  | X |  |  |  | X |  |  |  |  |  |  | X | X |  |  |  |  |  |  | X |
| Downing A, Forman D, Gilthorpe MS, Edwards KL, Manda SOM. (2008) | X |  |  |  |  |  |  | X | X |  |  |  | X |  |  |  | X |  |  |  | X |  |  |  |  |  |  | X | X |  |  |  |  |  |  | X |
| Draper GJ, Stiller CA, Cartwright RA, Craft AW, Vincent TJ. (1993) | X |  |  |  |  |  |  | X | X |  |  |  | X |  |  |  | X |  |  |  | X |  |  |  |  |  |  | X | X |  |  |  |  |  |  | X |
| Elliott P, Kleinschmidt I. (1997) | X |  |  |  |  |  |  | X | X |  |  |  | X |  |  |  | X |  |  |  | X |  |  |  |  |  |  | X |  |  | X |  |  |  |  | X |
| Ewings PD, Bowie C, Phillips MJ, Johnson SA. (1989) | X |  |  |  |  |  |  | X | X |  |  |  | X |  |  |  | X |  |  |  | X |  |  |  |  |  |  | X | X |  |  |  |  |  |  | X |
| Keenan TDL, Yeates D, Goldacre MJ. (2012) | X |  |  |  |  |  |  | X | X |  |  |  | X |  |  |  | X |  |  |  | X |  |  |  |  |  |  | X | X |  |  |  |  |  |  | X |
| Kinlen LJ, Dickson M, Stiller CA. (1995) | X |  |  |  |  |  |  | X | X |  |  |  | X |  |  |  | X |  |  |  | X |  |  |  |  |  |  | X | X |  |  |  |  |  |  | X |
| Lyons RA, Monaghan SP, Heaven M, Littlepage BN, Vincent TJ, Draper GJ. (1995) | X |  |  |  |  |  |  | X | X |  |  |  | X |  |  |  | X |  |  |  | X |  |  |  |  |  |  | X | X |  |  |  |  |  |  | X |
| Mangoud A, Hillier VF, Leck I, Thomas RW. (1985) | X |  |  |  |  |  |  | X | X |  |  |  | X |  |  |  | X |  |  |  | X |  |  |  |  |  |  | X | X |  |  |  |  |  |  | X |
| McKinney PA, Ironside JW, Harkness EF, Arango JC, Doyle D, Black RJ. (1994) | X |  |  |  |  |  |  | X | X |  |  |  | X |  |  |  | X |  |  |  | X |  |  |  |  |  |  | X | X |  |  |  |  |  |  | X |
| Muir K, Rattanamongkolgul S, Smallman-Raynor M, Thomas M, Downer S, Jenkinson C. (2004) |  |  | X |  |  |  |  | X |  |  | X |  |  | X |  |  |  |  | X |  | X |  |  |  |  |  |  | X | X |  |  |  |  |  |  | X |
| Musah A, Gibson JE, Leonardi-Bee J, Cave MR, Ander EL, Bath-Hextall F. (2013) | X |  |  |  |  |  |  | X | X |  |  |  | X |  |  |  | X |  |  |  | X |  |  |  |  |  |  | X | X |  |  |  |  |  |  | X |
| Rafiq M, Hayward A, Warren-Gash C, Denaxas S, Gonzalez-Izquierdo A, Lyratzopoulos G, et al. (2019) | X |  |  |  | X |  |  |  | X |  |  |  | X |  |  |  | X |  |  |  | X |  |  |  | X |  |  |  | X |  |  |  | X |  |  |  |
| Rait G, Horsfall L. (2020) | X |  |  |  | X |  |  |  | X |  |  |  | X |  |  |  | X |  |  |  | X |  |  |  | X |  |  |  | X |  |  |  | X |  |  |  |
| Renshaw C, Ketley N, Møller H, Davies EA. (2010) | X |  |  |  |  |  |  | X | X |  |  |  | X |  |  |  | X |  |  |  | X |  |  |  |  |  |  | X | X |  |  |  |  |  |  | X |
| Roberts RJ, Steward J, John G. (2003) | X |  |  |  |  |  |  | X | X |  |  |  | X |  |  |  | X |  |  |  | X |  |  |  |  |  |  | X | X |  |  |  |  |  |  | X |
| Saleh GM, Desai P, Collin JRO, Ives A, er, Jones T, et al. (2017) | X |  |  |  |  |  |  | X | X |  |  |  | X |  |  |  | X |  |  |  | X |  |  |  |  |  |  | X | X |  |  |  |  |  |  | X |
| Sans S, Elliott P, Kleinschmidt I, Shaddick G, Pattenden S, Walls P, et al. (1995) | X |  |  |  |  |  |  | X | X |  |  |  | X |  |  |  | X |  |  |  | X |  |  |  |  |  |  | X | X |  |  |  |  |  |  | X |
| Sehmer EAJ, Hall GJ, Greenberg DC, O'Hara C, Wallingford SC, Wright KA, et al. (2014) | X |  |  |  |  |  |  | X | X |  |  |  | X |  |  |  | X |  |  |  | X |  |  |  |  |  |  | X |  |  | X |  |  |  |  | X |
| Shack L, Jordan C, Thomson CS, Mak V, Møller H. (2008) | X |  |  |  |  |  |  | X | X |  |  |  | X |  |  |  | X |  |  |  | X |  |  |  |  |  |  | X | X |  |  |  |  |  |  | X |
| Stark JM, Black RJ, Brewster DH. (2007) | X |  |  |  |  |  |  | X | X |  |  |  | X |  |  |  | X |  |  |  | X |  |  |  |  |  |  | X | X |  |  |  |  |  |  | X |
| Steward J, John G. (2001) | X |  |  |  |  |  |  | X | X |  |  |  | X |  |  |  | X |  |  |  | X |  |  |  |  |  |  | X | X |  |  |  |  |  |  | X |
| Taib BG, Oakley J, Dailey Y, Hodge I, Wright P, du Plessis R, et al. (2018) | X |  |  |  |  |  |  | X | X |  |  |  | X |  |  |  | X |  |  |  | X |  |  |  |  |  |  | X | X |  |  |  |  |  |  | X |
| Toledano MB, Jarup L, Best N, Wakefield J, Elliott P. (2001) |  |  | X |  |  |  |  | X |  |  | X |  |  | X |  |  |  |  | X |  | X |  |  |  |  |  |  | X | X |  |  |  |  |  |  | X |
| Wheeler BW, Kothencz G, Pollard AS. (2013) | X |  |  |  |  |  |  | X | X |  |  |  | X |  |  |  | X |  |  |  | X |  |  |  |  |  |  | X | X |  |  |  |  |  |  | X |
| Wilkinson P, Thakrar B, Shaddick G, Stevenson S, Pattenden S, on M, et al. (1997) | X |  |  |  |  |  |  | X | X |  |  |  | X |  |  |  | X |  |  |  | X |  |  |  |  |  |  | X | X |  |  |  |  |  |  | X |
| Muller P, Woods L, Walters S. (2020) | X |  |  |  |  |  |  | X | X |  |  |  | X |  |  |  | X |  |  |  | X |  |  |  |  |  |  | X | X |  |  |  |  |  |  | X |
| McNally RJ AR, Cairns DP, Eden OB, Birch JM. (2003) | X |  |  |  |  |  |  | X | X |  |  |  | X |  |  |  | X |  |  |  | X |  |  |  |  |  |  | X | X |  |  |  |  |  |  | X |
| McNally RJ, Wakeford, R., James, P. W., Basta, N. O., Alston, R. D., Pearce, M. S., & Elliott, A. T. (2016) | X |  |  |  |  |  |  | X | X |  |  |  | X |  |  |  | X |  |  |  | X |  |  |  |  |  |  | X | X |  |  |  |  |  |  | X |
| dos Santos Silva I SA. (1993) |  |  | X |  |  |  |  | X |  |  | X |  |  | X |  |  |  |  | X |  | X |  |  |  |  |  |  | X | X |  |  |  |  |  |  | X |
| Richardson S, Abellan JJ, Best N. (2006) |  |  | X |  |  |  |  | X |  |  | X |  |  | X |  |  |  |  | X |  |  |  | X |  |  |  |  | X | X |  |  |  |  |  |  | X |
| Liao W, Coupland CAC, Innes H, Jepsen P, Matthews PC, Campbell C, The DeLIVER consortium, Barnes E, Hippisley-Cox J. (2023) | X |  |  |  |  |  |  | X | X |  |  |  | X |  |  |  | X |  |  |  | X |  |  |  |  |  |  | X | X |  |  |  |  |  |  | X |

Notes: Y = Yes; N = No; U = Unknown; NA = Not Applicable

# SUPPLEMENTARY FILE 7

PRIMA FLOWCHART

**Identification of studies via other methods**

**Identification of studies via databases and registers**

Studies removed *before screening*:

Duplicate records removed

(*n* =1,093)

Studies identified through database searching (*n* = 10,210)

WOS/SSCI: 4,583

MEDLINE: 4,672

SocINDEX: 40

CINAHL: 915

Records identified from:

Handsearching (*n* = 5)

Search strategy tests (*n* = 6)

**Identification**

Studies excluded

(*n* = 9,060)

Studies screened

(*n* = 9,117)

Studies not retrieved

(*n* = 6)

Studies sought for retrieval

(*n* = 11)

**Screening**

Studies not retrieved

(*n* = 6)

Studies sought for retrieval

(*n* = 57)

Studies excluded:

Ineligible outcomes (*n* = 3)

Ineligible methods (*n* = 1)

Studies assessed for eligibility

(*n* = 51)

Studies excluded:

(*n* = 0)

Studies assessed for eligibility

(*n* = 5)

**Included**

Studies included in review

(*n* = 52)

# SUPPLEMENTARY FILE 8

DESIGNS OF THE STUDIES INCLUDED IN THE SYSTEMATIC MAPPING REVIEW

# SUPPLEMENTARY FILE 9

SUMMARY TABLE

| **Reference list ID** | **Study author(s) and year** | **Data source** | **Geographical approach** | **Study’s objective(s)** | **Study design** | **Observation period** | **Study area(s)** | **Level of analyses** | **Number of cancer types** | **Types of cancers** | **Potential risk factors** | **Methods** | **Presentation of main results** |
| --- | --- | --- | --- | --- | --- | --- | --- | --- | --- | --- | --- | --- | --- |
| 45 | Mangoud et al., 1985 | North Western Regional Cancer Registry | Spatial distribution and time trend analyses | To analyse all pairs of patients by the distances between their homes and the intervals between the dates when they presented using Knox's test for space-time interaction. | Ecological study | 1962-1976 | England (Greater Manchester) | Counties | One cancer type | Blood and Lymphoid cells (Hodgkin’s disease) | Demographic factors (age); closeness in space and time | Cluster analysis (Knox's test for space-time interaction) | Only tables |
| 1 | Ewings et al., 1989 | South Western Regional Cancer Registry | Effect of proximity from potential risk sites | The incidence of leukaemia and non-Hodgkin's lymphoma in young people (aged under 25) living in a predefined area around the nuclear power station at Hinkley Point, Somerset, was examined for the period 1959-86 by using cancer registry data. | Ecological study | 1959-1986 | England (Hinkley Point, Somerset) | Multi-level | One cancer type | Blood and Lymphoid Cells (Leukaemia and non-Hodgkin's lymphoma <25yrs) | Environmental factors (nuclear installation) | Regression models (1-sided cumulative Poisson probability) | Only tables |
| 13 | Alexander et al., 1990 | The Leukaemia Research Fund (LRF) Data Collection Study | Effect of proximity from potential risk sites | The present study analyses incidence of leukaemias in  five diagnostic subgroups for the purpose of testing for association with two predefined ward characteristics, socio-economic status and proximity to estuaries. | Ecological study | 1984-1986 | England and Wales  (22 counties) | Wards | One cancer type | Blood and Lymphoid Cells  (Leukaemia <85yrs) | Socioeconomic factors (socioeconomic status); Environmental factors (estuarine wards) | Regression models (Poisson regression) | Plots and tables |
| 49 | Youngson et al., 1991 | North West Regional Cancer Registry; Yorkshire Cancer Registry; Leukaemia Research Fund's Centre for Clinical Epidemiology | Effect of proximity from potential risk sites | A population-based case control study of adult haematological malignancy and distance from, and magnetic fields associated with, overhead (OH) power lines has been carried out in the North West and Yorkshire regions of England. | Case-control | 1983-1995 | England  (North West and Yorkshire Regional Health Authorities) | health districts | One cancer type | Blood and Lymphoid Cells  (Leukaemia and non-Hodgkin's lymphoma >14yrs) | No statistically significantly raised OR's or trend (Environmental factors: proximity to powerlines) | Regression models (Logistic regression) | Only tables |
| 35 | Alexander et al., 1992 | Yorkshire Regional Children’s Tumour Registry; Northern Region Children's Malignant Disease Registry | Effect of proximity from potential risk sites | The objective was to compare residential histories of cases and controls and in particular to determine whether case children had lived in the same place at the same time more often than controls. | Case-control | 1974-1988 | England (West Cumbria, North Humberside, Gateshead) | Local Authorities | One cancer type | Blood and Lymphoid Cells  (Childhood leukaemia and Childhood non-Hodgkin lymphoma 0-14yrs) | residential proximity to areas with high incidence rates | Nearest neighbour computation; Cluster analysis (Cuzick-Edwards test) | Only tables |
| 14 | Craft et al., 1993 | Northern Region Children's Malignant Disease Registry; Manchester Children's Tumour Registry; Mersey Regional Cancer Registry | Spatial distribution and time trend analyses | To determine whether the seeming excess of childhood leukaemia and lymphoma identified in Seascale, Cumbria, UK, remains unusual when put into a wider context. The wider context taken for this study when compared with that presented to the Black committee, has been both geographical, to include the north western and part of the Mersey regions as well as the northern health region; temporal, extending the time period to 1985; has increased the age of cases considered to 24 years; and has considered all cancers as well as subtypes. | Ecological study | 1968-1985 | England (Greater Manchester, Tyne and Wear, Cleveland, Cumbria, Durham, Lancashire, and Northumberland administrative counties) | Wards | Multiple cancer type | Blood and Lymphoid Cells (Leukaemia and non-Hodgkin lymphoma <25yrs); Brain | Environmental factors (nuclear installation) for acute lymphoblastic leukaemia and non-Hodgkin's lymphoma | Regression models (Poisson probability ranking) | Only tables |
| 36 | dos Santos Silva, 1993 | National Cancer registry; Office of Population Censuses and Surveys | Spatial distribution and time trend analyses | The present study utilises data from the England and Wales National Cancer Registry and national mortality files to examine recent time trends and geographical distribution of thyroid cancer in the country, and to consider potential risk factors underlying them. | Cohort | 1962-1984 | England and Wales (all counties forming England and Wales) | Local Authorities | One cancer type | Thyroid | Demographic factors (sex and age). Associations found in counties in the North and rural areas | Odds ratios calculation | Plots and maps |
| 2 | Draper et al., 1993 | National Registry of Childhood Tumours; Northern Region Children's Malignant Disease Registry; Manchester Children's Tumour Registry; Northern Region Cancer Registry; North Western Regional Cancer Registry; Hospital and pathology department records | Effect of proximity from potential risk sites | To reappraise the epidemiological findings reported by the Black Advisory Group concerning a possible excess of malignant disease, particularly of childhood acute lymphoid leukaemia and non-Hodgkin lymphomas, in the vicinity of the Sellafield nuclear installation, and to determine whether any excess of malignant disease had occurred among people aged 0-24 years in the area in the years after the Black report-that is, from 1984 to 1990. | Ecological study | 1963-1990 | England (Cumbria) | Multi-level | Multiple cancer type | Blood and Lymphoid Cells (Leukaemia, non-Hodgkin lymphoma and Hodgkin disease <75yrs); Brain; Other (all other malignant diseases) | Environmental factors (nuclear installation) for lymphoid leukaemia and non-Hodgkin lymphomas | Incidence rates comparison (study areas and national data) | Only tables |
| 23 | Bhopal, et al., 1994 | Local general practices | Effect of proximity from potential risk sites | To determine whether there was excess ill health in people living near a coking works, and if so whether it was related to exposure to coking works' emissions. | Ecological study | 1986-1989 | England (South Tyneside) | Range of distances | Multiple cancer type | Lung; Genito urinary; Digestive; Blood and Lymphoid Cells (Lymphatic haematological); Lip; Oral cavity; Pharynx; Skin; Other and unspecified sites | No major and discernible impact (Environmental factors: proximity to coking works) | Regression models (log-linear modelling / GLIM) | Only tables |
| 52 | McKinney et al, 1994 | Scottish Cancer Registration Scheme; Scottish Morbidity Record for Inpatients; UK National Registry of Childhood Tumours | Spatial distribution and time trend analyses | The two aims of this paper are to assess the completeness and accuracy of the original cancer registration data for childhood CNS tumours and, for the validated data set, to describe the incidence and broad geographical distribution in Scotland. | Ecological study | 1975-1990 | Only Scotland (all health boards forming Scotland) | health board areas | Multiple cancer type | Brain, Central Nervous System | Socioeconomic factors (socioeconomic status) for brain and CNS tumours | Estimation of average annual percentage changes in incidence | Plots, tables and maps |
| 3 | Kinlen et al., 1995 | National Registry | Effect of proximity from potential risk sites | The authors examined the incidence of childhood leukaemia and non-Hodgkin's lymphoma near non-nuclear construction projects in rural Britain over the past 50 years. | Others (longitudinal study) | 1945-1993 | England and Scotland (Areas within 10 km of relevant sites, and the highland counties of Scotland with many hydroelectric schemes in comparison with Sellafield nuclear site) | Multi-level | One cancer type | Blood and Lymphoid Cells (Childhood Leukaemia and Childhood non-Hodgkin lymphoma <15 yrs) | Environmental factors (industrial and hydroelectric sites) | Observed/Expected calculation | Only tables |
| 48 | Lyons et al., 1995 | Welsh Cancer Registry | Effect of proximity from potential risk sites | To determine whether there was an increased incidence of leukaemias and lymphomas in young people aged less than 25 years in the locality of a petrochemical plant at Baglan Bay, South Wales. | Ecological study | 1974-1991 | Wales (South Wales) | Enumeration districts | One cancer type | Blood and Lymphoid Cells (Leukaemia and Lymphomas <25 yrs) | Incidence not significantly greater than normal (Environmental factors: proximity to petrochemical plants) | Observed/Expected calculation; Regression models (Poisson) | Only tables |
| 4 | Sans et al., 1995 | Office of Population Censuses and Surveys; The Information and Statistics Division of the Scottish Health Service | Effect of proximity from potential risk sites | To study incidence and mortality of leukaemias, cancer of the larynx, and other cancers near the petrochemical plant at Baglan Bay, in response to local concerns of an alleged cluster of cancers in the vicinity. | Ecological study | 1974-1984 | Wales (South Wales) | Multi-level | Multiple cancer type | Laryngeal; Blood and Lymphoid Cells (Leukaemia all ages) | Results may have been chance findings (Environmental factors: proximity to petrochemical works) | Stone's tests | Plots and tables |
| 24 | Dolk et al., 1997 | Office of National Statistics; Information and Statistics Division of the Scottish Health Service | Effect of proximity from potential risk sites | To investigate whether residence near a transmitter is associated with increased risk of adult leukaemia, skin melanoma, bladder cancer, childhood leukaemia and brain cancer. | Ecological study | 1974-1986 | Great Britain (selected areas near high power frequency modulation (FM) radio and television (TV) transmitters in Great Britain) | Range of distances | Multiple cancer type | Skin; Bladder; Blood and Lymphoid Cells (adult Leukaemia); Brain | Weak evidence of increased risk (Environmental factors: exposure to high power transmitters) | Observed/Expected calculation; Stone’s tests | Only tables |
| 25 | Dolk et al., 1997 | Office of National Statistics; Information and Statistics Division of the Scottish Health Service | Effect of proximity from potential risk sites | To investigate the incidence of selected cancers in the vicinity of Sutton Coldfield radio and television transmitter. | Ecological study | 1974-1986 | England (West Midlands) | Range of distances | Multiple cancer type | Blood and Lymphoid Cells (Leukaemia, Multiple myeloma, non-Hodgkin lymphoma >15 yrs); Brain; Central Nervous System; Skin; eye; Breast; Lung; Colon and Rectal; Stomach; Bladder; Prostate. | Environmental factors (radio and television transmitter) for leukaemias | Stone's tests; Pothoff-Whittinghill test; Observed/Expected calculation | Only tables |
| 26 | Elliott & Kleinschmidt, 1997 | Small Area Health Statistics Unit; ICI Chemicals and Polymers; World Register | Effect of proximity from potential risk sites | To investigate whether any non-occupational neighbourhood cases occurred near vinyl chloride sites | Ecological study | 1979-1986 (England, Wales); 1975-1987 (Scotland) | Great Britain (selected areas near vinyl chloride site in Great Britain) | Range of distances | One cancer type | Liver | No confirmed exposed cases (Environmental factors: proximity to vinyl chloride sites) | Number of cases in each range distance from the potential risk site | Plots and tables |
| 27 | Wilkinson et al., 1997 | Small Area Health Statistics Unit | Effect of proximity from potential risk sites | To examine the incidence and mortality of cancer near the Pan Britannica Industries factory, Waltham Abbey, after reports of a possible cluster of all cancers and brain cancer in the vicinity. | Ecological study | 1977-1989 (cancer incidence) | England (Waltham Abbey) | Range of distances | Multiple cancer type | Brain; respiratory organs; Pancreas; Stomach; Skin | Inconsistent evidence (Environmental factors: proximity to pesticide factory) | Observed/Expected calculation; Stone’s tests | Tables and maps |
| 32 | Badrinath et al., 1999 | East Anglian cancer registry | Effect of proximity from potential risk sites | To test further the hypothesis that there is a significant excess of leukaemia excluding chronic lymphoid leukaemia in estuarine wards, as identified by Alexander et al. (1990) | Ecological study | 1981– 1994 | England (East Anglian region) | postcode districts | One cancer type | Blood and Lymphoid Cells (Leukaemia >14 yrs) | Environmental factors (proximity to estuaries) | Observed/Expected calculation | Only tables |
| 46 | Cartwright et al., 1999 | Data Collection Study for registering haematological malignancies | Spatial distribution and time trend analyses | This paper carefully examines recently acquired descriptive data for possible clues for further investigation. | Ecological study | 1984–1993 | England (North England: West Yorkshire, North Yorkshire and the former Humberside, Lancashire and Cumbria; and Southwest England: South and West Regional NHS Office area of Hampshire, Isle of Wight, Dorset, Devon, Gloucestershire, Avon, Somerset, Wiltshire and Cornwall) | Counties | One cancer type | Blood and Lymphoid Cells (Multiple myeloma 0-84 yrs) | Demographic factors (sex and age); year of diagnosis | Regression models (Poisson regression; Linear regression) | Only tables |
| 28 | UK Childhood Cancer Study Investigators, 2000 | UK Childhood Cancer Study; Questionnaire | Effect of proximity from potential risk sites | To investigate whether proximity to electricity supply equipment or magnetic field exposure calculated from distance, load and other circuit information is associated with an increased risk of childhood cancer. | Case-control | 1 April 1992 to December 1996 (For leukaemias in England and Wales); 1 April 1992 to December 1995 (non-Hodgkin lymphomas in England and Wales); 1 April 1992 to December 1994 (Other malignancies in England and Wales); 1 January 1991 to December 1994 (Scotland) | Great Britain (study covering the whole of England, Scotland and Wales) | Range of distances | Multiple cancer type | Blood and Lymphoid Cells (Leukaemia and non-Hodgkin lymphoma 0-14 yrs); other malignancies | No evidence found (Environmental factors: proximity to powerlines) | Regression models (Logistic regression) | Plots and tables |
| 15 | Steward & John, 2001 | Welsh Cancer Intelligence and Surveillance Unit | Effect of proximity from potential risk sites | this ecological study explores the hypotheses that childhood cancer rates are increased by living near the coast of Wales, especially in the north, and in particular near estuaries and mud-flats. | Ecological study | 1985-1994 | Wales (Wales coastline) | Wards | Multiple cancer type | Blood and Lymphoid Cells (Childhood Leukaemia 0-14 yrs); Brain | No evidence found (Environmental factors: proximity to coastline) | Regression models (Poisson regression); Stone’s tests | Plots and tables |
| 16 | Toledano, 2001 | UK Small Area Health Statistics Unit | Spatial distribution and time trend analyses | The aim of this study was to assess temporal trends and analyse spatial variations in testicular cancer at a small area level across Great Britain. | Ecological study | 1975-1991 (Spatial trends); 1974-1991 (Temporal trends) | Great Britain (ward level across Great Britain) | Wards | One cancer type | Testicular cancer | socioeconomic factors (socioeconomic status) | Regression models (Poisson regression; Bayesian hierarchical modelling; Extended hierarchical model) | Plots, tables and maps |
| 17 | McNally et al., 2003 | Manchester Children’s Tumour Registry | Spatial distribution and time trend analyses | This study examined the geographical distribution of the incidence of acute leukaemia and lymphoma using Manchester Children’s Tumour Registry (MCTR) data 1976–2000. | Ecological study | 1976-2000 | England (North West region: Greater Manchester and Lancashire) | Wards | One cancer type | Blood and Lymphoid Cells (Childhood Leukaemia and Childhood Lymphomas 0-14 yrs) | Environmental factors (population density); socioeconomic factors (unemployment; household overcrowding) | Regression models (Poisson regression; Extra-Poisson variation) | Only tables |
| 18 | Roberts et al., 2003 | Wales Cancer Registry; Welsh Cancer Intelligence & Surveillance Unit Cancer Intelligence database | Effect of proximity from potential risk sites | The authors investigated claims by a campaigning group of a cancer cluster associated with a local cement works. | Ecological study | 1974-1989; 1985-1994 | Wales (Mold) | Wards | Multiple cancer type | Breast; Brain; Stomach; Colon and Rectal; Oesophagus; Thyroid; Lung; Respiratory cancers; Soft tissue sarcoma; Blood and Lymphoid Cells (non-Hodgkin lymphoma); | No evidence of increased incidence (Environmental factors: exposure to cement works) | Observed/Expected calculation | Only tables |
| 19 | Muir et al., 2004 | Trent Cancer Registry | Spatial distribution and time trend analyses | This study examines the spatial distribution of breast cancer incidence in Lincolnshire and Leicestershire, and its association with the application of selected pesticides. | Ecological study | 1989-1991 | England (Lincolnshire and Leicestershire) | Wards | One cancer type | Breast | Not found consistent associations (Environmental factors: pesticide application) | Correlation analysis (Spatial autocorrelation Black–White BW join-count statistic); Correlation analysis (Moran I coefficient); Regression models (Linear regression) | Tables and maps |
| 29 | Draper et al., 2005 | National Cancer Registration System; UK Children’s Cancer Study Group; National Registry of Childhood Tumours | Effect of proximity from potential risk sites | To determine whether there is an association between distance of home address at birth from high voltage power lines and the incidence of leukaemia and other cancers in children in England and Wales. | Case-control | 1962-1995 | England and Wales | Grid reference | Multiple cancer type | Blood and Lymphoid Cells (Childhood Leukaemia 0-14 yrs); Brain; Central Nervous System; Other diagnoses | Results may be due to chance or confounding (Environmental factors: proximity to high voltage powerlines) | Regression models (Logistic regression) | Only tables |
| 5 | Conway et al., 2006 | UK cancer registries | Spatial distribution and time trend analyses | This study aimed to determine whether the incidence of oral cancer is continuing to rise in the UK and if this varies geographically. | Others (descriptive epidemiological study) | 1990-1999 | United Kingdom (12 UK cancer registries) | Multi-level | Multiple cancer type | Lip; Mouth or oral cavity; Oropharynx | Demographic factors (sex and age). Significant associations found in North of England, Northern Ireland, Scotland and Wales for oral cancers | Regression models (Poisson regression) | Plots and tables |
| 31 | Edwards et al., 2006 | Local hospitals cancer registry lists, clinical databases and records | Effect of proximity from potential risk sites | A study was undertaken to investigate whether prolonged residence close to heavy industry is associated with lung cancer among women in Teesside. | Case-control | “last 20 years” | England (Teesside) | Exposure zones | One cancer type | Lung | Environmental factors (heavy industry) | Regression models (Logistic regression; Stepwise regression; Fractional polynomial regression) | Only tables |
| 6 | Stark et al., 2007 | Scottish Cancer Registry | Effect of proximity from potential risk sites | To investigate allegations of an excess risk of leukaemia among children living near the Solway Firth coast of Dumfries and Galloway Health Board area in Scotland, UK. | Ecological study | 1975-2002 | Scotland (Solway coast of Dumfries and Galloway Health Board area) | Multi-level | One cancer type | Blood and Lymphoid Cells (Childhood Leukaemia 0-14 yrs) | No statistically significant evidence (Environmental factors: proximity to estuaries) | Incidence estimation (standardised incidence ratios); Observed/Expected calculation | Only tables |
| 20 | Downing et al., 2008 | Northern & Yorkshire Cancer Registry database | Spatial distribution and time trend analyses | To model jointly the incidence rates of six smoking related cancers in the Yorkshire region of England, to explore the patterns of spatial correlation amongst them, and to estimate the relative weight of smoking and other shared risk factors for the relevant disease sites, both before and after adjustment for socioeconomic background | Ecological study | 1983-2003 | England (Yorkshire region) | Wards | Multiple cancer type | Oesophagus; Stomach; Pancreas; Lung; Kidney; Bladder | Socioeconomic factors (area deprivation for lung and stomach cancers); Environmental factors (urban areas for lung and stomach cancers); Lifestyle/behaviour factors (smoking for lung and stomach cancers; body weight/obesity for oesophagus cancer; diet/alcohol consumption for oesophagus cancer) | Regression models (Shared-component model - BYM) | Plots, tables and maps |
| 39 | Shack et al., 2008 | United Kingdom association of Cancer Registries | Spatial distribution and time trend analyses | The authors analysed the socioeconomic variations in the incidence of breast, lung and cervical cancer and malignant melanoma of the skin for England, and regionally and by age. | Ecological study | 1998-2003 | England ( English Government Office Regions) | Regions | Multiple cancer type | Breast; Lung; Cervix and Uterus; Skin | Demographic factors (age for lung cancer); Socioeconomic factors (deprivation status for lung, cervical, malignant melanoma and breast cancer); Significant associations found in NW and SW for cervical cancer; east midlands for breast cancer | Incidence estimation (age-standardised incidence rates); Rate ratios; Estimation of additional/fewer cases (based on deprivation groups); Observed/Expected calculation | Plots, tables and maps |
| 33 | Renshaw et al., 2010 | Thames Cancer Registry database | Spatial distribution and time trend analyses | The objective was to investigate trends in the epidemiology and survival of multiple myeloma for South East England. They also explored geographical variation in incidence across primary care trusts. | Others (cross-sectional) | 1985-2004 | England (South East) | Primary Care Trusts | One cancer type | Blood and Lymphoid Cells (Multiple myeloma all ages) | Demographic factors (sex and age) | Incidence estimation (age-standardised incidence rates) | Plots and maps |
| 7 | Keenan et al., 2012 | English national hospital episode statistics; Oxford record linkage study | Spatial distribution and time trend analyses | The aim was to report on trends over time and geographical variation in rates of uveal melanoma in England. | Ecological study | 1979-2010 | England (English Government Office Regions) | Multi-level | Multiple cancer type | Malignant neoplasm of choroid; Malignant neoplasm of ciliary body/iris; Skin | Demographic factors (sex and age); Socioeconomic factors (socioeconomic status) for uveal melanomas | Estimation of person-based admission rate | Plots, tables and maps |
| 21 | Bithell et al., 2013 | National Registry of Childhood Tumours | Effect of proximity from potential risk sites | It is the primary purpose of this paper to report the use of cases and controls collected by the National Registry of Childhood Tumours (NRCT) to determine the risk of leukaemia among children born close to a nuclear power plant (NPP) and further to investigate the risk of leukaemia and non-Hodgkin lymphoma (LNHL) with respect to residence at diagnosis by comparing the addresses of affected children with those suffering from other cancers. | Case-control | 1962–2007 | Great Britain (selected areas near nuclear installations in Great Britain) | Wards | One cancer type | Blood and Lymphoid Cells (Childhood Leukaemia and Childhood non-Hodgkin lymphoma <15 yrs) | Not statistically significant risk found (Environmental factors: proximity to nuclear power plants) | Regression models (Logistic regression) | Only tables |
| 40 | Elliott et al., 2013 | National Cancer Registry Data | Effect of proximity from potential risk sites | The authors conducted a case-control study to investigate risks of adult cancers in relation to distance and extremely low-frequency magnetic fields from high-voltage overhead power lines using National Cancer Registry Data in England and Wales, 1974-2008. | Case-control | 1974-2008 | England and Wales (selected areas near high-voltage overhead power lines) | Regions | Multiple cancer type | Blood and Lymphoid Cells (Leukaemia 15-74 yrs); Brain; Central Nervous System; Skin; Breast | No meaningful excess risks (Environmental factors: proximity to high voltage powerlines) | Regression models (Logistic regression); Correlation analysis (Pearson’s correlation) | Plots and tables |
| 8 | Musah et al., 2013 | The Health Improvement Network database | Spatial distribution and time trend analyses | To determine the variation in Basal cell carcinoma (BCC) throughout the U.K. | Others (population-based study) | 2004-2010 | United Kingdom (the whole of the U.K., in its constituent countries and principalities, and in each English SHA region) | Multi-level | One cancer type | Skin | Socioeconomic factors (socioeconomic status) | Regression models (Poisson regression; Stratified Poisson multivariable analyses) | Plots, tables and maps |
| 37 | Wheeler et al., 2013 | UK Association of Cancer Registries | Spatial distribution and time trend analyses | This study investigates the geography of non-melanoma skin cancer (NMSC) in England, and ecological associations with three widespread environmental hazards: radon, arsenic and ultraviolet radiation from the sun. | Ecological study | 2006-2008 | England (English Government Office Regions) | Local Authorities | One cancer type | Skin | Environmental factors (area-mean bright sunshine hours and area-mean radon concentration) | Regression models (Linear regression) | Plots, tables and maps |
| 38 | Abdulrahman et al., 2014 | Welsh Cancer Intelligence and Surveillance Unit | Spatial distribution and time trend analyses | To provide an up-to-date analysis of time trends in incidence, geographical distribution, survival and mortality from breast cancer in Wales. | Ecological study | 1985-2012 | Wales (22 local authority areas in Wales) | Local Authorities | One cancer type | Breast | Demographic factors (female). Higher incidence rates in rural areas | Regression models (Poisson regression) | Plots and tables |
| 30 | Bunch et al., 2014 | UK National Registry of Childhood Tumours | Effect of proximity from potential risk sites | This paper presents risk analyses for residential distance at birth from overhead high-voltage powerlines, which may be of aetiological interest in its own right, not just as a surrogate for the magnetic fields produced by the powerlines. They present risk analyses in relation to calculated magnetic fields separately. | Case-control | 1962–2008 | Great Britain (selected areas near overhead high- voltage power lines) | Grid references | Multiple cancer type | Blood and Lymphoid Cells (Childhood Leukaemia <15 yrs); Brain; Central Nervous System; Other solid tumours | Environmental factors (overhead high voltage powerlines) for leukaemias | Regression models (Logistic regression) | Plots and tables |
| 9 | Bunch et al., 2014 | Northern Region Young Persons’ Malignant Disease Registry; National Registry of Childhood Tumours; Office for National Statistics; Collaborators at Newcastle University; National Health Service Information Centre; Central Health Register Inquiry System; General Register Office for Scotland; Information Services Division (ISD) (Scotland) | Effect of proximity from potential risk sites | The authors aimed to update previous analyses relating to areas close to nuclear installations in Seascale and Dounreay by considering data from an additional 16 years of follow-up. | Cohort | 1991–2006 around either Sellafield or Dounreay; 1950 and 2006, while resident close to either installation, irrespective of their subsequent place of residence within Great Britain | England and Scotland (Sellafield and Dounreay) | Multi-level | Multiple cancer type | Blood and Lymphoid Cells (Leukaemia, non-Hodgkin lymphoma and Hodgkin lymphoma 0-24 yrs); Brain; Central Nervous System; Skin; Upper respiratory tract tumours; Lower respiratory tract tumours; Gastrointestinal tract tumours; Breast; Genito urinary; Thyroid; Other solid tumours | No significantly increased risks (Environmental factors: proximity to nuclear installations) | Observed/Expected calculation; Incidence estimation (standardised incidence ratios); Incidence rates comparison | Only tables |
| 51 | Sehmer et al., 2014 | Royal Preston Hospital records; National Cancer Data Repository | Spatial distribution and time trend analyses | The authors investigated in detail a clinically suspected cluster of gliomas in Lancashire and South Cumbria in the North West region of England. | Ecological study | 2006-2010 | England (Lancashire and South Cumbria) | Middle Layer Super Output Area | One cancer type | Central Nervous System | Demographic factors (sex and age) | Examine the significance of association (Chi-square tests); Regression models (Discrete Poisson model - satscan) | Plots and tables |
| 41 | Brodbelt et al., 2015 | National Cancer Registration Service; Hospital Episode Statistics for England | Spatial distribution and time trend analyses | The aim of this report is to examine the incidence of patients with glioblastoma in England, and to assess the influence of gender, age, geographical region and treatment on outcome. | Ecological study | 2007-2011 | England (English Government Office Regions) | Regions | One cancer type | Central Nervous System | Demographic factors (age) | Incidence estimation (age-standardised incidence rates) | Plots and tables |
| 10 | McNally et al., 2016 | National Cancer Registry | Spatial distribution and time trend analyses | The aim of the present study was to make a preliminary examination of the long-term rates of  incidence of thyroid cancer in those groups of people living in areas of north-west England most contaminated by iodine-131 from the 1957 Windscale Fire who were young at the time of the accident. | Ecological study | 1974-2012 | England (Lancashire and Cumbria) | Multi-level | One cancer type | Thyroid | Results does not confirm association (Environmental factor: exposure to iodine-131 near nuclear reactor fire) | Incidence rates comparison (study areas and national data) | Plots and tables |
| 50 | Saleh et al., 2017 | National Cancer Data Repository - England | Spatial distribution and time trend analyses | The authors report the incidence of eyelid BCC for the period 2000–2010. | Cohort | 2000-2010 | England (English Government Office Regions) | English cancer registries | One cancer type | Skin | Demographic factors (sex and age) | Incidence estimation (crude incidence rates and standardised incidence rates) | Plots and tables |
| 11 | Taib et al., 2018 | UK Cancer Information Service | Spatial distribution and time trend analyses | The aim of this longitudinal study was to examine the distribution of head and neck cancer (HANC) disease burden across the region comparing it to national trends. | Others (longitudinal study) | 1998-2000; 2008-2010; 2009-2011 | England (Merseyside and Cheshire Network) | Multi-level | One cancer type | Head and Neck | Socioeconomic factors (income deprivation; percentage of households in poverty); Lifestyle/behaviour factors (prevalence of adult smokers; populations at high and increasing risk of alcohol-related harm) | Correlation analysis (Pearson’s correlation) | Plots, tables and maps |
| 43 | Rafiq et al., 2019 | UK Clinical Practice Research Datalink; Hospital Episode Statistics | Spatial distribution and time trend analyses | This nationwide study conducted over a 25-year period in the UK investigates variation in HL incidence by age, sex, region and deprivation to identify trends and high-risk populations for HL development. | Cohort | 1992-2016 | United Kingdom (Strategic Health Authorities in UK) | Strategic Health Authorities | One cancer type | Blood and Lymphoid Cells (Hodgkin's lymphoma all ages) | Socioeconomic factors (socioeconomic status). Significant association found in North East of England | Regression models (Poisson regression) | Plots and tables |
| 42 | Chambers et al., 2020 | National Cancer Registration and Analysis Service database | Spatial distribution and time trend analyses | The aim of this study was to determine temporal trends in incidence of colorectal cancer stratified by sex, anatomical subsite in the colorectum, SES and geographical region of England. | Cohort | 1974-2015 | England (English Government Office Regions) | Regions | One cancer type | Colon and Rectal | Demographic factors (age); Tumour site; Significant association found in southern regions of England | Regression models (Joinpoint regression) | Plots and maps |
| 47 | Muller et al., 2020 | Office for National Statistics; National bowel and lung cancer audit datasets; Routes to Diagnosis dataset; Hospital Episodes Statistics records | Spatial distribution and time trend analyses | The authors evaluate temporal and geographical changes in stage at diagnosis during 2008–2013 for colorectal, non-small cell lung, and ovarian cancers, using multiple imputation to minimise bias from missing data. The authors evaluate whether the number of patients diagnosed at stages I or II increased; whether geographic inequalities increased or decreased; and whether observed changes are associated with case-mix. | Others (population-based study) | 2008-2013 | England (English Government Office Regions) | Clinical Commissioning Groups | Multiple cancer type | Colon and Rectal; Lung; Ovarian | Not Applicable (assessed if geographic inequalities increased or decreased over the study period. It reduced for colorectal and ovarian cancer. For non-small cell lung cancer there is no evidence that they decreased. ) | Regression models (Logistic regression) | Plots and tables |
| 44 | Rait & Horsfall, 2020 | The Health Improvement Network (THIN) IQVIA™ Medical Research Data; UK Biobank data | Spatial distribution and time trend analyses | The authors estimated age-adjusted incidence rates for recorded lung cancer before (1998−2007) and after (2008−2018) the introduction of smoke-free legislation and they also explored the impact of geographic location, social deprivation and urbanicity. | Cohort | 1998-2018 | United Kingdom (Strategic Health Authorities in UK) | Strategic Health Authorities | One cancer type | Lung | Socioeconomic factors (socioeconomic status); Significant association found in North of England | Regression models (Poisson regression) | Plots and tables |
| 12 | Burton et al., 2022 | National Cancer Registration Dataset in England; Linked Hospital Episodes Statistics Admitted Patient Care | Spatial distribution and time trend analyses | to determine if there was regional variation across the 19 English Cancer Alliances in the following areas: (1) incidence and change in incidence of HCC over the study period, (2) cause of underlying liver disease, (3) route to diagnosis of HCC; (4) proportion receiving potentially curative treatments; (5) survival. | Ecological study | 2010-2016 | England (English Government Office Regions) | Multi-level | One cancer type | Liver | Socioeconomic factors (socioeconomic status) | Regression models (Logistic regression) | Tables and maps |
| 22 | Richardson, Abellan & Best, 2006 | Not informed | Spatial distribution and time trend analyses | To present a joint analysis of the spatio-temporal variation of the risks of two related diseases processes–male and female lung cancer incidence–in a region of England. By using joint modelling, they aimed to extract the risk factors common to both genders and to study their temporal evolution and whether there is evidence of a differential risk between men and women that goes beyond that expected on the basis of the major risk factor for lung cancer, smoking. | Ecological study | 1981-1999 | England (Yorkshire) | Wards | One cancer type | Lung | Demographic factors (sex); Lifestyle/behaviour factors (smoking patterns) | Regression models (hierarchically centred parameterization of models); Correlation analysis (CAR model - conditional autoregressive Gaussian distribution) | Plots, tables and maps |
| 34 | Liao et al., 2023 | QResearch database | Spatial distribution and time trend analyses | This study aims to understand the disparities in epidemiology and clinical pathways of primary liver cancer and identify the gaps for early detection and diagnosis of liver cancer in England | Cohort | 2008-2018 | England (English Government Office Regions) | General Practices | One cancer type | Liver | Demographic factors (sex, age and ethnicity); Socioeconomic factors (socioeconomic status); Significant association in North West, North East and West Midlands of England | Regression models (Cox regression; Logistic regression) | Plots and tables |

* Number of references according to the bibliographic reference list at the end of this Supplementary File document.

# SUPPLEMENTARY FILE 10

NUMBER OF ARTICLES SELECTED IN THE SYSTEMATIC MAPPING REVIEW ACCORDING TO PERIOD OF PUBLICATION

# SUPPLEMENTARY FILE 11

GEOGRAPHIC UNITS CONSIDERED IN THE INCLUDED STUDIES

| **Number of studies** | **Study reference** | **Geographic units** | **Description** |
| --- | --- | --- | --- |
| 12 studies | (1-12) | Multi-level | Analyses conducted using different geographic units (e.g.: at Government Office Region (GOR), LAs, county districts, civil parishes, wards). |
| 10 studies | (13-22) | Wards and electoral division | They are the spatial units used to elect local government councillors in metropolitan and non-metropolitan districts, unitary authorities and the London boroughs in England. Electoral wards and electoral divisions have a population counts of 7,900 in average in UK. |
| 6 studies | (23-28) | Range of distances | Distance from potential risk sources (e.g. radii from nuclear installations). |
| 2 studies | (29, 30) | Grid references | National Grid in England and Wales. That is, all 275 and 400 kV overhead lines (the highest voltages used) plus a small fraction of 132 kV lines, about 7000 km altogether. |
| 1 study | (31) | Exposure zones | Study authors defined exposure zones in their study according to distances from heavy industries. |
| 1 study | (32) | Postcode districts | Postcodes are alphanumeric references comprising an outward code of two-to-four characters (e.g. PO15) and an inward code of three characters (e.g. 5RR). Postcode districts are defined by the first four letters of a postcode (e.g. PO15) and may contain up to 100 addresses. |
| 1 study | (33) | Primary Care Trusts | A Primary Care Trust (PCT) is a legal entity, set up by order of the Secretary of State for Health and Social Care. It is a free-standing NHS body, performance managed by a Strategic Health Authority. |
| 1 study | (34) | General Practices | General practices form the cornerstone of primary care in England. They are for most people the first and most commonly-used point of access to the NHS. |
| 4 studies | (35-38) | Local Authority Level | Local Authority is an organisation responsible for a range of services for individuals and businesses. |
| 4 studies | (39-42) | Regions | There are nine English regions which were established in 1994 and they are the highest tier of sub-national division in England. |
| 2 studies | (43, 44) | Strategic Health Authorities | A Strategic Health Authority is an NHS organisation established to lead the strategic development of the local health service and manage Primary Care Trusts and NHS Trusts based on local accountability agreements. |
| 2 studies | (45, 46) | Counties | Counties are administrative structures set up across England and Wales, except for the Isles of Scilly, Greater London and the six metropolitan counties. |
| 1 study | (47) | Clinical Commissioning Group | Clinical Commissioning Groups were clinically-led statutory NHS bodies responsible for the planning and commissioning of health care services for their local area (2013-2022). |
| 1 study | (48) | Enumeration Districts | An Enumeration District is used across the United Kingdom for the purposes of census data collection. Enumeration districts are within Wards. |
| 2 studies | (49, 50) | English Cancer Registries/Health districts | When someone is diagnosed with cancer in the UK, information about the patient, cancer diagnosis and treatment is collected in a cancer registry. This is used to plan and improve health and care services. The hospital will usually give this information to the registry automatically. Local cancer registries feed into a national registry. |
| 1 study | (51) | Middle Layer Super Output Area | Middle layer Super Output Areas (MSOAs) comprise between 2,000 and 6,000 households and have a resident population usually between 5,000 and 15,000 persons. |
| 1 study | (52) | Health Board | NHS Scotland consists of 14 regional NHS Boards which are responsible for the protection and improvement of the population’s health and for the delivery of frontline healthcare services and eight Special NHS Boards which support the regional NHS Boards by providing a range of important specialist and national services. |

# SUPPLEMENTARY FILE 12

STUDY AREAS OF INCLUDED ARTICLES

# SUPPLEMENTARY FILE 13

POTENTIAL RISK FACTORS IDENTIFIED IN STUDIES FOCUSED ON THE NORTH WEST OF ENGLAND

| **Number of studies** | **Study reference** | **Potential risk factors** |
| --- | --- | --- |
| 3 | (45, 46, 51) | Demographic |
| 3 | (2, 14, 17) | Environmental |
| 2 | (11, 17) | Socioeconomic |
| 1 | (11) | Lifestyle/behaviour |
| 2 | (35, 46) | Others |

# SUPPLEMENTARY FILE 14

POTENTIAL RISK FACTORS IDENTIFIED IN STUDIES.

| **Number of studies** | **Study reference** | **Potential risk factors** |
| --- | --- | --- |
| 14 | (5, 7, 22, 33, 34, 36, 38, 39, 41, 42, 45, 46, 50, 51) | Demographic factors |
| 13 | (7, 8, 11-13, 16, 17, 20, 34, 39, 43, 44, 52) | Socioeconomic factors |
| 12 | (1-3, 13, 14, 17, 20, 25, 30-32, 37) | Environmental factors |
| 6 | (11, 20, 22, 35, 42, 46) | Others |

# SUPPLEMENTARY FILE 15

METHODS OUTLINE (FOR SPATIAL DISTRIBUTION AND TIME TREND ANALYSES)

**Recent studies (published from 2010 to present) – One cancer type considered**

| **Reference list ID** | **Study author(s) and year** | **Study title** | **Methods** | **Outline** | **Strengths highlighted** | **Limitations highlighted** |
| --- | --- | --- | --- | --- | --- | --- |
| 33 | Renshaw et al., 2010 | Trends in the incidence and survival of multiple myeloma in South East England 1985-2004 | Incidence estimation (age-standardised incidence rates) | The authors explored geographical variation in the incidence of multiple myeloma at the level of individual primary care trust (1995-2004). They presented the age-standardised incidence rates for each area and grouped into quintiles | By calculating age-standardised incidence rates, the authors could account for age differences across the Primary care trusts.  The authors do not discuss strengths regarding methods. | The authors noted that case ascertainment may have varied across different PCTs, which could affect the reliability of the incidence rates reported. The South Thames Haematology Register only covered part of the area during the study period, potentially leading to incomplete data for some regions. This study did not investigate the influence of other factors (e.g. ethnicity) on incidence and survival. The authors do not discuss limitations regarding the methods employed – including the absence of confunder and risk factors. |
| 8 | Musah et al., 2013 | Regional variations of basal cell carcinoma incidence in the U.K. using The Health Improvement Network database  (2004–10) | Regression models (Poisson regression; Stratified Poisson multivariable analyses) | This study provides novel estimates for regional incidence rates across the U.K. • They also provide novel estimates for levels of socioeconomic deprivation in the U.K. A Poisson multivariable regression model was used to examine the effects of all factors (i.e. calendar year of diagnosis, socioeconomic deprivation and regions) on the incidence of BCC adjusted for sex and age groups. Stratified Poisson multivariable analyses were used to determine whether associations between all factors and the incidence of BCC were modified by sex, while controlling for age groups. For secondary analyses, they further used stratified models by age groups to assess calendar years as a continuous variable in order to determine the average change (per year) in incidence of BCC. IR ratios (IRRs) were estimated with 95% confidence intervals (CIs). | This study uses the largest sample size of incident cases of BCC compared with previous research conducted in the U.K. Due to large sample size, findings are unlikely to be by chance. Also, the data were obtained from a national database and prospectively recorded by general practitioners, thus excluding the possibility of recording or recall bias in either exposure or outcome. Results for country-level IRs are consistent with previous studies showing escalating rates in England, Northern Ireland, Scotland and Wales. The authors do not discuss strengths regarding methods. | The major limitation is the inability to account for important factors such as history of sun exposure during childhood and adolescence (frequency of sunburns and overseas holidays), latitudinal position (proximity to the equator), settings of occupation (indoor, mixed or outdoor) and skin type (fair, white or freckled skin). In addition, the authors were unable to classify adults according to subtypes of BCC. The authors do not discuss limitations regarding the methods employed and therefore no mention on the potential presence of spatial dependent effect. |
| 37 | Wheeler et al., 2013 | Geography of non-melanoma skin cancer and ecological associations with environmental risk factors in England | Regression models (Linear regression) | Linear regression models were used to assess associations between age-/sex-standardised rates of NMSC and arsenic, radon and bright sunshine hours, with adjustment for area deprivation and outdoor occupation prevalence. NMSC mapped at Local Authority level. | The comprehensive geographical extent of the data and the large population considered. As environmental risk factors often have relatively weak effects, but affect large populations, large data sets are valuable in providing appropriate scale and sufficient statistical power. Although the regression models could not account for uncertainty in the standardised rates, confidence intervals and observed case counts published alongside the rates indicate that they are subject to relatively small standard error. This could be expected, given that they are 3-year aggregate rates of a relatively common disease for fairly large populations. | The findings are subject to the limitations of the study design and data available. These are aggregate data, and inferring individual risk from population-level associations invokes the ecological fallacy. The LA-level environmental variables may themselves be subject to error, as they are summary measures derived from finer resolution data. No mention on the potential presence of spatial dependent effect is made. |
| 38 | Abdulrahman et al., 2014 | Breast cancer in Wales: time trends and geographical distribution | Regression models (Poisson regression) | European age-adjusted standardised rates (EASR) were determined according to the standardised European population for the period 1985-2012.  Poisson regression model was fitted to assess temporal trends and rate ratios (RR) and 95% confidence intervals (CI) were determined and compared in relation to age, geographical distribution and mortality across time periods. | The authors acknowledge that the study has been facilitated by the availability of a robust cancer registry in Wales that has systematically obtained records over the past four decades.  They discuss the results by comparing with other studies, but they do not discuss strengths regarding methods. | The findings rely on the quality of the coded data in the database. Detailed histopathology coding was not available, which could have resulted in better insights into the trends of breast cancer in Wales.  The authors discuss the limitations on the aspect of the quality and detail of data used but not regarding the methods employed including failing in accounting for spatial dependent effects.  . |
| 51 | Sehmer et al., 2014 | Incidence of glioma in a northwestern region of England, 2006-2010 | Examine the significance of association (Chi-square tests); Regression models (Discrete Poisson model - satscan) | Chi-square tests were used to assess differences in distributions of age and sex of patients and differences in the histological subtypes, grades, and location of the tumours. The authors also examined the potential for spatial-temporal clustering of higher-than-expected incidence at MSOA level over the period 2001–2010. | The authors highlight as strength the population-based nature of the primary data and their recency.  They discuss the study results by comparing with other regional reports and also with the incidences for the North West region. The authors do not discuss strengths regarding methods. | Study weaknesses include a proportion of tumours in the National Brain Tumour Registry for England that were not specified as gliomas per se but rather as malignant brain tumours.  The authors discuss the limitations on the aspect of the quality and detail of data used. The authors do not discuss limitations regarding the methods employed including the lack of analyses on confounders and risk factors. |
| 41 | Brodbelt et al., 2015 | Glioblastoma in England: 2007-2011 | Incidence estimation (age-standardised incidence rates) | Annual European Age Standardised Incidence rates per 100,000 population were calculated for overall and age specific cohorts, using standard techniques. The standard population used is the 2013 European Standard Population. | Incidence data presented in the current report are affected by different coding practices and age variations, limiting valid comparisons.  The authors discuss their results by comparing with other countries and previous studies, but they do not discuss strengths regarding methods. | The absence of radiotherapy data and the failure to define linked chemotherapy type are weaknesses of this report. Quality of life data were not examined in the present study and might mitigate slight increases in life expectancy. Improved data quality currently being achieved will allow more searching analyses to be possible in the near future.  The authors do not discuss limitations regarding the methods employed in particular the lack of analyses on confunders and risk factors. |
| 10 | McNally et al., 2016 | A geographical study of thyroid cancer incidence in north-west England following the Windscale nuclear reactor fire of 1957 | Incidence rates comparison (study areas and national data) | Incidence rate ratios (IRRs), together with corresponding 95% confidence intervals (CIs) calculated by assuming that thyroid cancer incidence rates follow a Poisson distribution, were used to compare Cumbria, Lancashire, and Cumbria plus Lancashire, with the Rest of England providing reference rates. The assumption of Poisson distributed rates was examined by testing for overdispersion. | The authors do not discuss strengths regarding methods. | Difficulties of interpretation of the patterns of thyroid cancer IRRs are exacerbated by the highly significant extra-Poisson variation exhibited by the distributions. When appropriate adjustment is made to account for overdispersion, none of the IRRs differs significantly from 1.0. The extra-Poisson variation could be due to a non-uniform presence of major thyroid cancer risk factors, or shortcomings in the incidence data, or both.  The authors discuss other limitations of study focusing on interpretations of results and ecological fallacy, scale of analysis and compare the results by reviewing other studies assessing exposure to iodine-131. The authors do not discuss limitations regarding the methods employed, including the lack of analyses on confunders and risk factors. |
| 50 | Saleh et al., 2017 | Incidence of eyelid basal cell carcinoma in England: 2000–2010 | Incidence estimation (crude incidence rates and standardised incidence rates) | Age-standardised incidence rates per 100 000 population for eyelid basal cell carcinoma by age group and sex for 3-year cohorts, 2000–2010, England. The authors report crude incidence rate per 100 000 population of eyelid basal cell carcinoma by age and sex, 2000– 2010, England and Age-standardised incidence rates per 100 000 population for eyelid basal cell carcinoma, by sex, for three-year cohorts, 2000–2010, England. | The authors focused on discussion of results, but do not discuss strengths of methods used. | The authors discuss limitations regarding data registration and data quality. They state that During the period studied, there was regional variation in the registration of BCC. This may be due to a variety of factors relating to clinical practice for reporting new cases of eyelid BCC and cancer registry processes for registration, as well as demographic and lifestyle risk factors. The authors do not discuss limitations in methods, including the lack of analyses on confunders and risk factors. |
| 11 | Taib et al., 2018 | Socioeconomic deprivation and the burden of head and neck cancer—Regional variations of incidence and mortality in Merseyside and Cheshire, North West, England. | Correlation analysis (Pearson’s correlation) | Comparisons were made of the direct age-standardised incidence and mortality rates between the comparator regions and the LAs. Using England as a benchmark, 95% confidence intervals derived from UK Cancer Information Service allowed us to compare the confidence intervals of the estimates with non-overlapping confidence intervals being considered as statistically significant difference. Lifestyle and deprivation measures were plotted against direct age-standardised incidence and mortality rates to obtain correlation relationships as measured by Pearson’s correlation coefficients. | The authors focused on discussion of results, but do not discuss strengths of methods used. | This descriptive study is limited in that the authors cannot prove causation nor determine the effect size of the deprivation measures analysed. They could not comment on occupational exposures, HPV status, stage or histology at presentation, and educational status and access to healthcare facilities which may confound any correlation. They also acknowledge that confidence intervals in some cases may overlap and represent a significant difference between groups. Further, they assume linearity for the Pearson’s correlation coefficient when comparing indicators of deprivation against HANC incidence and mortality. The authors do not discuss limitations in methods including the lack of analyses on confunders and risk factors, and the risk that correlation may be affected by spatial dependence. |
| 43 | Rafiq et al., 2019 | Socioeconomic deprivation and regional variation in Hodgkin’s lymphoma incidence in the UK: a population-based cohort study of 10 million individuals. | Regression models (Poisson regression) | Poisson regression was used to model HL incidence rate ratios (IRRs) for region, deprivation, age and sex independently before adjusting for other variables. Deprivation was initially included as a categorical variable in the regression analysis to calculate IRRs and then subsequently the authors assessed for a linear trend by deprivation quintile, first by estimating the linear effect of deprivation using likelihood ratio tests, and then investigating departure from linearity by comparing models in which deprivation was added as a non-linear vs a linear term. In addition, incidence rates by deprivation were examined to see if any variation persisted after adjusting for trends in region, and vice versa to see if trends in region were observed after adjusting for deprivation as a categorical variable. Adjusted models were also adjusted for age and sex. | The main strengths of this study are that it is a large population-based study of more than 10 million individuals and has a long follow-up. HL is a relatively rare disease, and the sample size and follow-up length allow for smaller effect sizes and interactions that could be missed in smaller studies to be detected. A further advantage of this study was the use of CPRD data with regional information, linked to HES and deprivation data. The authors do not discuss strengths of methods used. | The main limitation of this study is that it did not have access to linked data from the UK National Cancer Registry (NCR), which can be considered to represent the gold standard for estimating HL incidence. Data was also not available on HL subtype and EBV positivity status, which would be informative for subgroup analysis to assess if trends in deprivation varied by histological group. Another limitation is the use of routinely collected data with potential misclassification of an individual’s deprivation group. Further studies are required to investigate the role of different factors in regional variation in UK HL incidence (e.g. environmental factors, regional differences in ethnicity and clustering of predisposing or protective genotypes). The authors don’t discuss limitations in methods, including the absence of any assessment of spatial dependence. |
| 42 | Chambers et al., 2020 | Demographic trends in the incidence of young-onset colorectal cancer: a population-based study. | Regression models (Joinpoint regression) | Temporal trends in age-specific incidence rates according to sex, anatomical subsite, index of multiple deprivation quintile and geographical region were analysed using Joinpoint regression. Joinpoint Regression Program was used to analyse the magnitude and direction of temporal trends in age-specific incidence rates according to sex, anatomical site, IMD quintile and geographical region. Age–period–cohort modelling was used to assess the independent effects of age, time period and cohort on colorectal cancer incidence rates. | The main strengths of this study are the size and completeness of the data set. Data were obtained from NCRAS, a nationally curated cancer registry, with 100 per cent complete data for 1974–2012 and 98⋅4 per cent complete data for 2013–2015. The authors do not discuss strengths of methods used. | Stage-specific data were not recorded routinely until 2012, so further analysis of incidence rate trends according to tumour stage was not possible. Data presented in this study are population-based and specific causal inferences cannot be made. In addition, IMD quintile and geographical region are group-level metrics and are unable to account for individual-level contextual effects that could have affected the association between these variables and colorectal cancer incidence rates. The role of environmental factors, such as diet, obesity, physical exercise and the gut microbiota, in the development of young-onset colorectal cancer is incompletely understood and requires further research. The authors do not discuss limitations in methods, and while they take into account temporal dependence, the spatial dependence is neglected. |
| 44 | Rait & Horsfall, 2020 | Twenty-year sociodemographic trends in lung cancer in non-smokers: A UK based cohort study of 3.7 million people | Regression models (Poisson regression) | The authors estimated age-adjusted incidence rates for recorded lung cancer before (1998−2007) and after (2008−2018) the introduction of smoke-free legislation using multivariable Poisson regression. They also explored the impact of geographic location, social deprivation and urbanicity. | The main advantage of a large-scale analysis of LCINS for a single nation or country, is the ability to understand any temporal changes in the context of demographic shifts and government legislation. The dataset derived from routine health records is representative of the UK suggesting this cohort is broadly representative of people reporting as non-smokers to their primary care physicians. The authors do not discuss strengths of methods used. | The study relies on self-reported smoking status to define this cohort. Further, the authors are relying on a GP interpretation of self-reported smoking status and the definition of a “never” smoker may differ across GPs and GP practices. The authors analysed lung cancer cases recorded in general practice.  These data have been shown to be accurate for chronic conditions but less so for acute conditions that present to hospitals. The sociodemographic and urbanicity variables are derived from the UK Census for 2001 and may not be as accurate for more recent time periods. These two variables are only available from the data provider as categorical variables and the mutually adjusted estimates should be interpreted with caution. UK Biobank participants are not representative of the general population and cannot be used to provide representative disease prevalence and incidence rates. The authors do not discuss limitations in methods including the lack of employment of spatial methods. |
| 12 | Burton et al., 2022 | Regional variations in hepatocellular carcinoma incidence, routes to diagnosis, treatment and survival in England | Regression models (Logistic regression) | Logistic regression models were used to explore variation in primary liver disease aetiology by Cancer Alliance, unadjusted and adjusted for age, sex, deprivation quintile and ethnicity. Odds ratios were converted to proportions and the p-value for difference between the 19 Cancer Alliances calculated using the likelihood ratio test. Results were displayed in choropleth maps (in quintiles) and in forest plots to show differences across regions. | The analysis was population-based and includes a large number of HCC patients nationally. It used high quality data from the national cancer registry with near complete population coverage meaning that extrapolation was not needed and consequently, selection biases were minimised. Highly trained cancer registration officers in NCRAS standardise cancer registrations across the country using multiple data sources, allowing accurate between region comparisons.  The authors do not discuss strengths of methods used. | There was insufficient data detail or completeness available to include cancer stage, synthetic liver function and performance status in these analyses. The majority of HCCs are diagnosed using radiological techniques with high specificity (rather than using histology) in line with EASL clinical guidelines, so a small proportion of non HCC tumours (e.g. neuroendocrine tumours or metastases or mixed HCC-cholangiocarcinoma), may have been included, as would be the case with any population-based study in a country with similar diagnostic practices. No individual-level measure of deprivation was available, and the ecological deprivation measure used, based on the income domain of the index of multiple deprivation allocated on the small area level, may not have accurately captured all socioeconomic differences, therefore the effect of socioeconomic deprivation on regional differences may have been under-estimated. The authors do not discuss limitations in methods, including the lack of employment of spatial methods. |
| 34 | Liao et al., 2023 | Disparities in care and outcomes for primary liver cancer in England during 2008–2018: a cohort study of 8.52 million primary care population using the QResearch database. | Regression models (Cox regression; Logistic regression) | Regression models were used to investigate factors associated with an incident diagnosis of liver cancer, emergency presentation, late stage at diagnosis, receiving treatments, and survival duration after diagnosis by subtype. The crude and European age-standardised incidence rates for the three liver cancer subtypes were calculated and plotted by sex, calendar year, and liver cancer subtypes. Cox regression was used to investigate whether patients’ sociodemographic characteristics (i.e. age groups, sex, ethnicity, socioeconomic deprivation using Townsend quintile as a proxy) and the ten geographical regions in England were associated with an incident diagnosis of the three liver cancer subtypes. Logistic regression was used to investigate the factors associated with (1) emergency presentation as route to cancer diagnosis compared with other routes and (2) late stage at diagnosis (Stages III or IV) compared with early stages (I or II), where patients with unknown stage were not included in the analysis. | This study has three key strengths. Firstly, the authors used a representative cohort consisting of approximately 20% of the whole English primary care population. Secondly, this paper reports up-to-date statistics of incidence and survival based on long periods of clinical follow-up, as well as comprehensive information on the milestone events in the clinical pathways of liver cancer in England, while most papers on cancer epidemiology only report the overall statistics of incidence and mortality by age groups and sex. Thirdly, this study also benefited from data linkage, which provided important information on cancer stage, histology, treatment modalities, and causes of death for primary liver cancer cases. The authors do not discuss strengths of methods used. | The authors did not investigate the aetiology (e.g. hepatitis B/C virus infection, alcohol, non-alcoholic fatty liver disease, etc.) of primary liver cancer, since this is not the primary aim of this study, but it is a limitation. there may be some coding errors in the “other specified/unspecified liver cancer” group, which could result in misclassification bias of either the outcomes or the associated features. Individual socioeconomic status is not available in EHR, Townsend score quintile was used as a proxy for material deprivation instead. However, this is a common practice in UK health research. The authors do not discuss limitations in methods including the lack of employment of spatial methods. |

# SUPPLEMENTARY FILE 16

SPATIAL STATISTICAL METHODS OUTLINE (FOR SPATIAL DISTRIBUTION AND TIME TREND ANALYSES)

**Past studies (from 1985 to 2009) – One cancer type considered**

| **Reference list ID** | **Study author(s) and year** | **Study title** | **Methods** | **Outline** | **Strengths highlighted** | **Limitations highlighted** |
| --- | --- | --- | --- | --- | --- | --- |
| 45 | Mangoud et al., 1985 | Space-time interaction in Hodgkin's disease in Greater Manchester | Cluster analysis (Knox's test for space-time interaction) | Knox's test for space-time interaction was applied to 922 cases of Hodgkin's disease which, according to the North Western Regional Cancer Registry, had presented in 1962-76 among the population of the region known before 1974 as the South East Lancashire Conurbation. | The present study yielded no evidence of a definite incubation period or of clustering specific to a particular histological subtype of HD. The authors focused on discussion of results, but do not discuss strengths of methods used. | One limitation is that the approach does not distinguish between excess pairs that are concentrated in the same locality (as the female child pairs were) and excess pairs that are scattered throughout the study area. Clearly, further work is needed to establish whether the excesses of close pairs observed in adults occur in particular localities.  The analysis is based on an index and therefore ignore spatial and temporal confunders. |
| 36 | dos Santos Silva, 1993 | Thyroid cancer epidemiology in England and Wales: time trends and geographical distribution | Odds ratios calculation | Age-adjusted odds ratios (Mantel & Haenszel, 1959) and test-based 95% confidence limits (Miettinen, 1976) for each county were calculated using the SAS statistical package (SAS Institute, 1988). The odds ratios were calculated in each sex for all ages combined, and also separately at younger older ages. Odds ratios were mapped using the Mapics computer program (Campbell & Nicholson, 1989). For display, odds ratios were divided between the highest and the lowest values into seven equally spaced intervals on a logarithmic scale. | The completeness of cancer registration is known to vary across England and Wales (Swerdlow et al., in press. Rates calculated using population estimates as denominators would therefore be biased. Instead, the authors calculated odds ratios to estimate the risk of thyroid cancer ('cases') relative to other cancer sites ('controls') in each county ('exposed') compared with the rest of the country ('non-exposed'). | Odds ratios will be unaffected by incompleteness or duplication of registration unless its degree is markedly dissimilar for different cancer sites. Even if there were very dissimilar completenesses by site, a low apparent risk of a tumour would be a much more plausible artefact than a high apparent risk, since the latter could only arise if there were great incompleteness for most other cancers but not for thyroid cancer (or if thyroid cancer registrations were grossly duplicated but registrations for other sites generally were not). The method is unadjusted for other confunders and risk factors. |
| 46 | Cartwright et al., 1999 | Epidemiology of multiple myeloma in parts of England, 1984–1993 | Regression models (Poisson regression; Linear regression) | standardized morbidity ratios (SMRs) were calculated and examined for evidence of geographical variation in incidence. Incidence rates were tested for evidence of trends over time. Simple plots of age- and sex-specific incidence rates against year of diagnosis were examined and Poisson regression models were fitted. Simple linear regression of directly standardized rates for each year of diagnosis was also carried out. | This case series is unique in several respects; in particular it is one of the largest specialist registry studies to be reported. It is certainly the largest series known to the authors which has been carefully verified and is dependent upon pathology records alone, and no other means of ascertainment of cases. It is also the only series carefully cross-checked with an independent dataset to enable estimates of completeness, overall, to be in excess of 96 per cent. The authors do not discuss strengths of methods used. | The comparison of rates with other registries is not exact in that the data in this study are censured at 85 years of age; it is not likely, however, that this would markedly increase the rates. It may be that registry data for multiple myeloma include some patients with monoclonal gammopathy of undetermined significance (MGUS) or those with raised paraprotein reports. Without further haematological or histopathological evidence, such cases would not be included in the DCS. This may explain some of the inflation of MM rates in the elderly when registry data are compared with the DCS. The authors do not discuss limitations in method including the exclusion of spatial dependence effect in the models. |
| 16 | Toledano, 2001 | Spatial variation and temporal trends of testicular cancer in Great Britain | Regression models (Poisson regression; Bayesian hierarchical modelling; Extended hierarchical model) | Poisson regression was used to examine for regional and socio-economic effects and Bayesian mapping techniques to analyse small-area spatial variability. This analysis takes no account of possible spatially structured variation in risk of testicular cancer, so the authors then extended the hierarchical model to produce estimates of both spatially neutral variation (leading to global smoothing of risk as before) and spatially structured variation (leading to local smoothing of risk). | This study is the first to examine both temporal trends and geographical variation of testicular cancer at both regional and small-area scales. The authors standardized the expected counts for this analysis by registry to account for possible differences between cancer registries in case ascertainment. | Within registries there may be local variations in ascertainment that the authors were not able to account for, and which would add to the observed variability at small area scale. Further difficulties in interpreting geographical variation include the long latency periods of this cancer and the effects of migration. |
| 17 | McNally et al., 2003 | Geographical and ecological analyses of childhood acute leukaemias and lymphomas in north-west England | Regression models (Poisson regression; Extra-Poisson variation) | The authors have analysed the data for the possible existence of spatial clustering. They have performed a test for the general presence of localized excesses, which is also known as extra-Poisson variation. Poisson regression was used to examine the relationship between incidence rates and small-area (census ward) population density, ethnic composition and deprivation index. | This is the first analysis to examine the geographical distribution of ALL by immunophenotype. The study has only been made possible by the availability of high-quality and consistent population-based diagnostic and residential address data. As ascertainment is close to 100%, there is no reason to suspect that there is any artefactual bias by small area of diagnosis. The authors do not discuss strengths of methods used | The ethnic composition of the ward is not necessarily related to characteristics of individual cases and should only be regarded as an ecological measurement. Likewise, population density and the deprivation scores are ward based and are not individual characteristics. Area-level data have been assigned to individual cases. Care should be exercised when using such grouped data to make inferences about individuals. The authors do not discuss limitations in methods, including the exclusion of spatial random effects. |
| 19 | Muir et al., 2004 | Breast cancer incidence and its possible spatial association with pesticide application in two counties of England | Correlation analysis (Spatial autocorrelation Black–White BW join-count statistic); Correlation analysis (Moran I coefficient); Regression models (Linear regression) | The Black–White (BW) join-count statistic and Moran I coefficient were  used to investigate the localized distribution of breast cancer. Linear regression techniques were applied to examine the association between the breast cancer incidence rate and pesticide application. | The authors focused on discussion of results, but do not discuss strengths of methods used. | In relation to locating breast cancer cases, the use of addresses at diagnosis may not represent the areas where the cases lived and were exposed to pesticides for the longest time. Another drawback of this study is that employing pesticide application in the areas might be a poor surrogate for actual exposure to pesticides. In relation to pesticide data, one could argue that information of pesticide application in 1991 might not be a good proxy for temporal lag time between exposure and development of breast cancer and, over time, levels of application of particular pesticides may have changed in a given geographical area. Thus, levels of application in 1991 may not have been the same as levels in the latent period. Simple linear regression also has methodological limitations. One of the drawbacks of this method is the problem of using aggregated data. That is, the information on pesticide application was collected for areal units and the data might not represent exposure in individuals. A further problem relates to the possible effects of known or unknown confounding factors, e.g. socio-economic factors. In further analyses that assessed whether there was any correlation between ward deprivation score and breast cancer incidence, no association was seen so this factor was not included in the modelling. |
| 22 | Richardson, Abellan & Best, 2006 | Bayesian spatio-temporal analysis of joint patterns of male and female lung cancer risks in Yorkshire (UK) | Regression models (hierarchically centred parameterization of models); Correlation analysis (CAR model - conditional autoregressive Gaussian distribution) | The authors present a joint analysis of the spatio-temporal variation of the risks of two related diseases processes–male and female lung cancer incidence–in a region of England. They used a Bayesian hierarchical model that splits the risk of disease into two spatio-temporal components: a shared component and a specific component that calibrates the differential between the two diseases. | The authors have illustrated how these models can be progressively elaborated by adding additional terms besides the main effects of disease, space and time. Second order interaction terms can be estimated and used to dissect interesting disease– space, disease–time and space–time patterns if the epidemiological context warrants it. They have also shown that it is important to consider the amount of information available in the data, which can be used to identify shared and specific patterns. One interesting feature of these models is the possibility to use the second order interaction terms to highlight unusual patterns. | The authors acknowledge potential limitations related to data quality issues and the challenge of hypothesizing specific risk factors (e.g. migration effects or the presence of a new hospice in an area), which could affect the interpretation and validity of the findings. The authors do not discuss limitations in methods. |

# SUPPLEMENTARY FILE 17

SPATIAL STATISTICAL METHODS OUTLINE (FOR SPATIAL DISTRIBUTION AND TIME TREND ANALYSES)

**Recent studies (from 2010 to Present) – Multiple cancer types considered**

| **Reference list ID** | **Study author(s) and year** | **Study title** | **Methods** | **Outline** | **Strengths highlighted** | **Limitations highlighted** |
| --- | --- | --- | --- | --- | --- | --- |
| 7 | Keenan et al., 2012 | Uveal melanoma in England: trends over time and geographical variation | Estimation of person-based admission rate | No recent study has examined trends in the rate of patients diagnosed with uveal melanoma in England or investigated geographical variation within England. The aims were to analyse trends over time and geographical variation in uveal melanoma. Geographical data were used to construct maps showing the person-based admission rate (first occurrence only) per100 000 resident population for each GOR or LA, expressed as average annual rates (uveal melanoma). The admission rate for each LA was plotted against its score for the index of multiple deprivation. In addition, the admission rate for each LA was plotted against the proportion of individuals in that LA according to ethnicity. Finally, a map was constructed showing the person-based admission rate (first occurrence only) by LA for cutaneous melanoma. | This study demonstrates that hospital data such as HES and ORLS are useful in the analysis of trends in uveal melanoma. The authors argue their incidence rate is similar to that in the published literature, as are their age and sex-specific incidence rates. Finally, one important advantage of linked hospital data is that it is possible to correlate individual patients with different operations or procedures.  The authors focused on discussion of results and highlight strengths of the data used, but do not discuss strengths of methods. | As with cancer registry data, hospital data rely on accurate coding and reporting of clinical information, for example, correct anatomical subtype of ocular melanoma.  One disadvantage of HES and ORLS data is that ICD categories do not currently encode histological information for uveal malignancies. HES datasets contain record linkage only from 1998; Therefore, they used ORLS data to complement HES data.  The authors reported some limitations on the data and its registration, but do not discuss limitations of methods, including justification of the absence of data modelling. |
| 47 | Muller et al., 2020 | Temporal and geographic changes in stage at diagnosis in England during 2008–2013: A population-based study of colorectal, lung and ovarian cancers | Regression models (Logistic regression) | Logistic regression was used to estimate temporal and geographical changes in early diagnosis adjusted for case mix using a multilevel model. The change in the odds of diagnosis at stages I or II between the two-year time periods was estimated using multilevel logistic regression models. The first set of models included only time period and CCG as explanatory variables. A second set of case-mix adjusted variables were fitted including these variables along with age, sex, CCI, tumour topography, and tumour morphology. | The authors highlight strengths in their methods for dealing with missing data and focused on discussing results. They used multiple imputation – a gold-standard approach to minimise bias in cases where a fraction of data is irretrievably missing - in estimates of national changes in stage at diagnosis. | The authors imputed missing stage information by assuming that it is missing randomly conditional on all the other information available, including on patient’s subsequent survival It is likely that this assumption is not entirely met, and that this approach reduced but did not eliminate bias.  Another restriction of this study is that data after reform of the NHS in 2013 were not available. This reform may have had a positive or negative impact on early diagnosis.  No justification for the use of an aspatial model is provided. |

# SUPPLEMENTARY FILE 18

SPATIAL STATISTICAL METHODS OUTLINE (FOR SPATIAL DISTRIBUTION AND TIME TREND ANALYSES)

**Past studies (from 1993 to 2009) – Multiple cancer types considered**

| **Reference list ID** | **Study author(s) and year** | **Study title** | **Methods** | **Outline** | **Strengths highlighted** | **Limitations highlighted** |
| --- | --- | --- | --- | --- | --- | --- |
| 14 | Craft et al., 1993 | Cancer in young people in the north of England, 1968-85: analysis by census wards | Regression models (Poisson probability ranking) | Analysis of cancer incidence by geographical area. Wards were ranked by cancer incidence and Poisson probability, using different population bases. | The authors focused on discussion of data and results, but do not discuss strengths of methods used. | The authors vaguely discuss limitations in methods and acknowledge that it is difficult to attach real significance to the absolute Poisson probability figure because of the problem of multiple significance testing. According to the authors, for a study such as this, the rank position of a ward is probably more important than the absolute Poisson probability value.  Spatial random effects were not included in the model. |
| 52 | McKinney et al, 1994 | Registration quality and descriptive epidemiology of childhood brain tumours in Scotland 1975-90 | Estimation of average annual percentage changes in incidence | Trends in incidence were examined by calculating age standardised rates, using the direct method and the world standard population. Incidence rates throughout are expressed per million years of childhood (0- 14 years) population. Average annual percentage changes in incidence were estimated by fitting regression lines to the logarithms of the age-standardised rates for the years 1975-90. Standardised registration ratios (SRRs) with 95% confidence intervals were used to compare incidence in the health board areas of Scotland. | The authors focused on discussion results and comparison with other studies, but do not discuss strengths of methods used. | The authors discuss limitation in data, specifically, regarding data registration and ascertainment. They mentioned misclassification of histological coding, although the influence of this on standard cancer registration statistics would be minimal. The authors also highlighted that ecological correlation studies always have limitations in that they characterise an area and not an individual with the disease. The authors do not discuss limitations of methods and no attempt to model the data is made. |
| 5 | Conway et al., 2006 | Incidence of oral and oropharyngeal cancer in United Kingdom (1990–1999) ––recent trends and regional variation | Regression models (Poisson regression) | Poisson regression models were used to assess the significance of trends in incidence after adjusting for age (5 year age groups) and sex. Variations in trends between cancer registries were assessed using Poisson regression to examine interactions between time and cancer registries. Poisson regression was also used to model the incidence trends, adjusting for any changes in the age profile of the population (using registry specific populations adjusted for age profile not EASR). Estimates of the percentage change from the first to the last year (and annually) in incidence over time were extracted from the models. | The authors focused on discussion results and comparison with other studies, but do not discuss strengths of methods used. | The authors mention limitation in data, such as under reporting of oral cancer by some UK cancer registries has previously been noted. Under ascertainment of data inevitably influences national incidence rates and in comparisons of this nature one needs to caution on errors arising thereof. They highlight that further examination and modelling of the data on pattern and type of alcohol consumption including binge drinking, and the interaction of alcohol with other lifestyle factors, diet and nutrition, viral infections, genetic determinants and socio-economic circumstances along with the smoking data is essential. The authors do not discuss limitations of methods used, including the potential presence of spatial dependence |
| 20 | Downing et al., 2008 | Joint disease mapping using six cancers in the Yorkshire region of England | Regression models (Shared-component model - BYM) | The joint analysis of the spatial variation in incidence used a Bayesian shared-component model. Three components were included to represent differences in smoking (for all six sites), bodyweight/ obesity (for oesophagus, pancreas and kidney cancers) and diet/alcohol consumption (for oesophagus and stomach cancers). | This study demonstrates the feasibility of joint disease modelling using data from six cancer sites. Smoothing allows us to see the inherent spatial patterns of cancer incidence as the variability or 'noise' has been removed. The estimates are more precise, and the associated CIs are smaller than those obtained without smoothing. Their study has shown that a joint model of the six cancer sites offers a great improvement over individual BYM models (as demonstrated by the DIC criteria). | The interpretation of the derived smoothed maps should be treated with caution because, although they are a great improvement over unsmoothed maps, there remain confounding factors, differences in population size, and area factors. In addition, the results may suffer from over- or under-estimation, due to the edge effects phenomenon. The authors used an adjacency matrix to ascertain the number of neighbours each ward had. However, some wards in the Yorkshire region border wards in other regions. Data were not available for other regions; thus, these wards have missing neighbours, which may introduce some bias when averaging rates or when estimating spatial effects as their variances are inversely related to the total number of areas. |
| 39 | Shack et al., 2008 | Variation in incidence of breast, lung and cervical cancer and malignant melanoma of skin by socioeconomic group in England | Incidence estimation (age-standardised incidence rates); Rate ratios; Estimation of additional/fewer cases (based on deprivation groups); Observed/Expected calculation | European age-standardised incidence rates (ASRs) were calculated by deprivation quintile, cancer site, Government Office Region (GOR), age group (under 65, 65 and over) and gender. Rate Ratios (RR) were calculated by region and age (under 65 and 65 and over) using the least deprived (quintile 1) as the baseline. The number (and percentage) of additional/fewer incident cases that would be expected if incidence rates in all deprivation groups was the same as those in the least deprived was estimated. The difference between the observed and expected number of cases for socioeconomic group was calculated to provide an estimate of the burden of cancer should these inequalities change. | The authors focused on discussion results and comparison with other studies, but do not discuss strengths of methods used. | The authors discuss limitations in data collection by registration stating that there is probably some regional variation due to differences in data collection and timeliness, although this is unlikely to explain the magnitude of differences. Completeness of registration information, such as treatment and stage, is known to be less complete for deprived patients. These variations would only influence the results of this study if a patients' diagnosis date were changed, and even in this case very little impact would be expected due to grouping of years, however this is unlikely due to the high quality of registry data. Ascertainment of lung and breast cancers are known to be high although regional variations in ascertainment of malignant melanoma have been shown for the early 1990s with late-stage tumours more likely to be registered. Increased use of hospital admission system data in the late 1990s and 2000s would be expected to improved ascertainment, although no direct comparison of this has been published. However, the authors do not discuss limitations in methods used, including the absence of data modelling |

References

1. Ewings PD, Bowie C, Phillips MJ, Johnson SA. Incidence of leukaemia in young people in the vicinity of Hinkley Point nuclear power station, 1959-86. BMJ (Clinical research ed). 1989;299(6694):289-93.

2. Draper GJ, Stiller CA, Cartwright RA, Craft AW, Vincent TJ. Cancer in Cumbria and in the vicinity of the Sellafield nuclear installation, 1963-90. BMJ (Clinical research ed). 1993;306(6870):89-94.

3. Kinlen LJ, Dickson M, Stiller CA. Childhood leukaemia and non-Hodgkin's lymphoma near large rural construction sites, with a comparison with Sellafield nuclear site. BMJ (Clinical research ed). 1995;310(6982):763-8.

4. Sans S, Elliott P, Kleinschmidt I, Shaddick G, Pattenden S, Walls P, et al. Cancer incidence and mortality near the Baglan Bay petrochemical works, South Wales. Occupational and environmental medicine. 1995;52(4):217-24.

5. Conway DI, Stockton DL, Warnakulasuriya KAAS, Ogden G, Macpherson LMD. Incidence of oral and oropharyngeal cancer in United Kingdom (1990-1999) -- recent trends and regional variation. Oral oncology. 2006;42(6):586-92.

6. Stark JM, Black RJ, Brewster DH. Risk of leukaemia among children living near the Solway coast of Dumfries and Galloway Health Board area, Scotland, 1975-2002. Occupational and environmental medicine. 2007;64(1):66-8.

7. Keenan TDL, Yeates D, Goldacre MJ. Uveal melanoma in England: trends over time and geographical variation. The British journal of ophthalmology. 2012;96(11):1415-9.

8. Musah A, Gibson JE, Leonardi-Bee J, Cave MR, Ander EL, Bath-Hextall F. Regional variations of basal cell carcinoma incidence in the U.K. using The Health Improvement Network database (2004-10). The British journal of dermatology. 2013;169(5):1093-9.

9. Bunch KJ, Vincent TJ, Black RJ, Pearce MS, McNally RJQ, McKinney PA, et al. Updated investigations of cancer excesses in individuals born or resident in the vicinity of Sellafield and Dounreay. British journal of cancer. 2014;111(9):1814-23.

10. McNally RJ, Wakeford, R., James, P. W., Basta, N. O., Alston, R. D., Pearce, M. S., & Elliott, A. T. A geographical study of thyroid cancer incidence in north-west England following the Windscale nuclear reactor fire of 1957. Journal of Radiological Protection. 2016;36(4).

11. Taib BG, Oakley J, Dailey Y, Hodge I, Wright P, du Plessis R, et al. Socioeconomic deprivation and the burden of head and neck cancer-Regional variations of incidence and mortality in Merseyside and Cheshire, North West, England. Clinical otolaryngology : official journal of ENT-UK ; official journal of Netherlands Society for Oto-Rhino-Laryngology & Cervico-Facial Surgery. 2018;43(3):846-53.

12. Burton A, Balach, rakumar VK, Driver RJ, Tataru D, Paley L, et al. Regional variations in hepatocellular carcinoma incidence, routes to diagnosis, treatment and survival in England. British journal of cancer. 2022;126(5):804-14.

13. Alexander FE, Cartwright RA, McKinney PA, Ricketts TJ. Leukaemia incidence, social class and estuaries: an ecological analysis. Journal of public health medicine. 1990;12(2):109-17.

14. Craft AW, Parker L, Openshaw S, Charlton M, Newell J, Birch JM, et al. Cancer in young people in the north of England, 1968-85: analysis by census wards. Journal of Epidemiology & Community Health. 1993;47(2):109-15.

15. Steward J, John G. An ecological investigation of the incidence of cancer in Welsh children for the period 1985-1994 in relation to residence near the coastline. JOURNAL OF THE ROYAL STATISTICAL SOCIETY SERIES A-STATISTICS IN SOCIETY. 2001;164:29-43.

16. Toledano MB, Jarup L, Best N, Wakefield J, Elliott P. Spatial variation and temporal trends of testicular cancer in Great Britain. British journal of cancer. 2001;84(11):1482-7.

17. McNally RJ AR, Cairns DP, Eden OB, Birch JM. Geographical and ecological analyses of childhood acute leukaemias and lymphomas in north-west England. British Journal of Haematology. 2003;123(1):60-5.

18. Roberts RJ, Steward J, John G. Cement, cancers and clusters: an investigation of a claim of a local excess cancer risk related to a cement works. Journal of Public Health Medicine. 2003;25(4):351-7.

19. Muir K, Rattanamongkolgul S, Smallman-Raynor M, Thomas M, Downer S, Jenkinson C. Breast cancer incidence and its possible spatial association with pesticide application in two counties of England. Public health. 2004;118(7):513-20.

20. Downing A, Forman D, Gilthorpe MS, Edwards KL, a SOM. Joint disease mapping using six cancers in the Yorkshire region of England. INTERNATIONAL JOURNAL OF HEALTH GEOGRAPHICS. 2008;7.

21. Bithell JF, Murphy MFG, Stiller CA, Toumpakari E, Vincent T, Wakeford R. Leukaemia in young children in the vicinity of British nuclear power plants: a case-control study. British journal of cancer. 2013;109(11):2880-5.

22. Richardson S, Abellan JJ, Best N. Bayesian spatio-temporal analysis of joint patterns of male and female lung cancer risks in Yorkshire (UK). Statistical Methods in Medical Research. 2006;15:385-407.

23. Bhopal RS, Phillimore P, Moffatt S, Foy C. Is living near a coking works harmful to health? A study of industrial air pollution. Journal of epidemiology and community health. 1994;48(3):237-47.

24. Dolk H, Elliott P, Shaddick G, Walls P, Thakrar B. Cancer incidence near radio and television transmitters in Great Britain. II. All high power transmitters. American journal of epidemiology. 1997;145(1):10-7.

25. Dolk H, Shaddick G, Walls P, Grundy C, Thakrar B, Kleinschmidt I, et al. Cancer incidence near radio and television transmitters in Great Britain. I. Sutton Coldfield transmitter. American journal of epidemiology. 1997;145(1):1-9.

26. Elliott P, Kleinschmidt I. Angiosarcoma of the liver in Great Britain in proximity to vinyl chloride sites. Occupational and environmental medicine. 1997;54(1):14-8.

27. Wilkinson P, Thakrar B, Shaddick G, Stevenson S, Pattenden S, on M, et al. Cancer incidence and mortality around the Pan Britannica Industries pesticide factory, Waltham Abbey. Occupational and environmental medicine. 1997;54(2):101-7.

28. Childhood cancer and residential proximity to power lines. UK Childhood Cancer Study Investigators. British journal of cancer. 2000;83(11):1573-80.

29. Draper G, Vincent T, Kroll ME, Swanson J. Childhood cancer in relation to distance from high voltage power lines in England and Wales: a case-control study. BMJ (Clinical research ed). 2005;330(7503):1290.

30. Bunch KJ, Keegan TJ, Swanson J, Vincent TJ, Murphy MFG. Residential distance at birth from overhead high-voltage powerlines: childhood cancer risk in Britain 1962-2008. British Journal of Cancer. 2014;110(5):1402-8.

31. Edwards R, Pless-Mulloli T, Howel D, Chadwick T, Bhopal R, Harrison R, et al. Does living near heavy industry cause lung cancer in women? A case-control study using life grid interviews. Thorax. 2006;61(12):1076-82.

32. Badrinath P, Day NE, Stockton D. Geographical clustering of acute adult leukaemia in the East Anglian region of the United Kingdom: a registry-based analysis. Journal of epidemiology and community health. 1999;53(5):317-8.

33. Renshaw C, Ketley N, Møller H, Davies EA. Trends in the incidence and survival of multiple myeloma in South East England 1985-2004. BMC cancer. 2010;10:74.

34. Weiqi Liao CACC, Hamish Innes, Peter Jepsen, Philippa C. Matthews, Cori Campbell, Eleanor Barnes, Emma Culver, Roman Fischer, Julia Hippisley-Cox, Hamish Innes, William L. Irving, Peter Jepsen, Matt Kelly, Paul Klenerman, Weiqi Liao, Derek Mann, Aileen Marshall, Philippa C. Matthews, Michael Pavlides, Rory J.R. Peters, Elisabeth Pickles, James Robineau, Benjamin Schuster-Böckler, Chunxiao Song, Jeremy Tomlinson, Christopher Welberry, Eleanor Barnes, Julia Hippisley-Cox. Disparities in care and outcomes for primary liver cancer in England during 2008–2018: a cohort study of 8.52 million primary care population using the QResearch database. eClinicalMedicine. 2023;59.

35. Alexander FE, McKinney PA, Moncrieff KC, Cartwright RA. Residential proximity of children with leukaemia and non-Hodgkin's lymphoma in three areas of northern England. British journal of cancer. 1992;65(4):583-8.

36. dos Santos Silva I SA. Thyroid cancer epidemiology in England and Wales: time trends and geographical distribution. British Journal of Cancer. 1993;67(2):330-40.

37. Wheeler BW, Kothencz G, Pollard AS. Geography of non-melanoma skin cancer and ecological associations with environmental risk factors in England. British Journal of Cancer. 2013;109(1):235-41.

38. Abdulrahman GOJ. Breast cancer in Wales: time trends and geographical distribution. Gland surgery. 2014;3(4):237-42.

39. Shack L, Jordan C, Thomson CS, Mak V, Møller H. Variation in incidence of breast, lung and cervical cancer and malignant melanoma of skin by socioeconomic group in England. BMC Cancer. 2008;8:271-.

40. Elliott P, Shaddick G, Douglass M, de Hoogh K, Briggs DJ, Toledano MB. Adult cancers near high-voltage overhead power lines. Epidemiology (Cambridge, Mass). 2013;24(2):184-90.

41. Brodbelt A, Greenberg D, Winters T, Williams M, Vernon S, Collins VP. Glioblastoma in England: 2007-2011. European journal of cancer (Oxford, England : 1990). 2015;51(4):533-42.

42. Chambers AC, Dixon SW, White P, Williams AC, Thomas MG, Messenger DE. Demographic trends in the incidence of young-onset colorectal cancer: a population-based study. The British journal of surgery. 2020;107(5):595-605.

43. Rafiq M, Hayward A, Warren-Gash C, Denaxas S, Gonzalez-Izquierdo A, Lyratzopoulos G, et al. Socioeconomic deprivation and regional variation in Hodgkin's lymphoma incidence in the UK: a population-based cohort study of 10 million individuals. BMJ open. 2019;9(9):e029228.

44. Rait G, Horsfall L. Twenty-year sociodemographic trends in lung cancer in non-smokers: A UK-based cohort study of 3.7 million people. Cancer epidemiology. 2020;67:101771.

45. Mangoud A, Hillier VF, Leck I, Thomas RW. Space-time interaction in Hodgkin's disease in Greater Manchester. Journal of epidemiology and community health. 1985;39(1):58-62.

46. Cartwright RA, Gilman EA, Nicholson P, Allon D. Epidemiology of multiple myeloma in parts of England, 1984-1993. Hematological oncology. 1999;17(1):31-8.

47. Muller P, Woods L, Walters S. Temporal and geographic changes in stage at diagnosis in England during 2008-2013: A population-based study of colorectal, lung and ovarian cancers. Cancer epidemiology. 2020;67:101743.

48. Lyons RA, Monaghan SP, Heaven M, Littlepage BN, Vincent TJ, Draper GJ. Incidence of leukaemia and lymphoma in young people in the vicinity of the petrochemical plant at Baglan Bay, South Wales, 1974 to 1991. Occupational and environmental medicine. 1995;52(4):225-8.

49. Youngson JH, Clayden AD, Myers A, Cartwright RA. A case/control study of adult haematological malignancies in relation to overhead powerlines. British journal of cancer. 1991;63(6):977-85.

50. Saleh GM, Desai P, Collin JRO, Ives A, er, Jones T, et al. Incidence of eyelid basal cell carcinoma in England: 2000-2010. The British journal of ophthalmology. 2017;101(2):209-12.

51. Sehmer EAJ, Hall GJ, Greenberg DC, O'Hara C, Wallingford SC, Wright KA, et al. Incidence of glioma in a northwestern region of England, 2006-2010. Neuro-oncology. 2014;16(7):971-4.

52. McKinney PA, Ironside JW, Harkness EF, Arango JC, Doyle D, Black RJ. Registration quality and descriptive epidemiology of childhood brain tumours in Scotland 1975-90. British journal of cancer. 1994;70(5):973-9.
