# Supplementary material for: Systematic mapping review of statistical methods applied to the relationships between cancer diagnosis and geographical level factors in UK
Source: BMJ Open. 2025 Jul 6;15(7):e098379. doi: 10.1136/bmjopen-2024-098379 (PMC12230968; doi:10.1136/bmjopen-2024-098379)
Supplement: Supplementary file 2 [file bmjopen-2024-098379.reviewer_comments.pdf]

PEER REVIEW HISTORY

BMJ Open publishes all reviews undertaken for accepted manuscripts. Reviewers are asked to complete a checklist review form and are provided with free text boxes to elaborate on their assessment. These free text comments are reproduced below.

This paper was submitted to another journal from BMJ but declined for publication following peer review. The authors addressed the reviewers' comments and submitted the revised paper to BMJ Open. The paper was subsequently accepted for publication at BMJ Open.

ARTICLE DETAILS

Title (Provisional)

A Systematic Mapping Review of statistical methods applied to the relationships between cancer diagnosis and geographical level factors in UK

Authors

Mendes, Jessica Andretta; Keegan, Thomas; Jones, Lisa; Atkinson, Peter M; Sedda , Luigi

---

VERSION 1 - REVIEW

**Reviewer**	**1**
**Name**	**Reviewer 1**
**Affiliation**	
**Date**	**28-Oct-2024**
**COI**	**No competing interest**

Comments not published pending reviewer's consent

VERSION 2 - REVIEW

**Reviewer**	**2**
**Name**	**Sokale , Itunu**
**Affiliation**	**Baylor College of Medicine**
**Date**	**14-Feb-2025**
**COI**	**None**

Thank you for the opportunity to review this work. The review article identified an important gap in the methodology of studies of cancers and geographic variables. Only a minor edit in the abstract line 13.

Please change "one of the worst areas in UK due to high cancer incidence" to "UK areas with disproportionately high cancer incidence"

**Reviewer**	**3**
**Name**	**Schündeln, Michael**
**Affiliation**	**University Hospital Essen, Pediatric oncology**
**Date**	**19-Feb-2025**
**COI**	**None**

Thank you to the editors for the opportunity to review this well written and thought-provoking manuscript of an important subject.

The authors of this study were interested in the published research analyzing the spatial distribution of various cancer types in association with demographic, environmental, behavioural and/or socioeconomic factors in the UK. Particular attention was focused to the north of the UK.

The initial goal was to conduct a systematic review, which would have involved an in-depth analysis focused onthe cancer incidence in association with the various factors while considering specific statistical methods. In fact, the authors found, that the heterogeneity between the studies included did not support such a review. As a result, they had to compromise by presenting a systematic mapping review, which provides a broad overview of research trends and gaps without presenting numerical results or addressing these specific questions.

The authors found that most of the studies were regression analyses covering only one cancer type. Only a few studies resembled spatially explicit models and therefore most studies did not account for interactions between cancer incidence and their determinants on a geographical scale. The authors conclude that there is an urgent need to use spatial statistics as this could contribute towards greater understanding of the spatial patterns of cancer distribution the existence of risk factors and possible prevention strategies.

General Remarks:

- The manuscript covers an interesting and important research topic.
- The manuscript is highly readable, employing clear and concise language.
- All sections of the manuscript are coherently structured
- The introduction effectively establishes the relevance of the research topic
- The methods are thoroughly described
- PRISMA Statement has been included
- All relevant source data are included and summarized
- The analyses are presented in an accessible manner
- It appears the article has already been reviewed and an extensive and mostly plausible rebuttal has been elaborated. A number of points have been changed and discussed in the manuscript following this review.
-

Specific Remarks:

- As the authors pointed out, numerical/quantitative statistics have not been performed in the review. It would have been interesting, though to get some more detail on the quantitative findings those studies, which include quantitative statistics. This would be important for the discussion regarding the kind of research and methods that need to be undertaken for a better understanding of the subject.
- In the same regard, the quantitative results of the spatially explicit models should be presented briefly. This could lead to a discussion on which methods may be employed in further studies (e.g. Besag-Newell, Bayesian Besag-York-Mollié, spatial scan statistics or other statistical methods for areal-cross-sectional data).
- The authors discuss only the work of Arcaya et al., which reviews a similar subject for the United States. Here, too, one main limitation is the lack of publications applying spatial statistical models. It would be interesting to learn whether such research has been performed successfully elsewhere.

Summary:

In summary, the authors present an interesting and relevant study. As they state this is the first systematic mapping review about this topic for the northern UK. It is surprising that no more comprehensive spatial statistics studies have been published so far.

The main point of the work is the implicit appeal that more effort should be dedicated to research in spatial statistics concerning the determinants of cancer development, especially with new computational methods, big data, etc., at hand, this could be a major opportunity to gain deeper insights into risk factors and possible prevention strategies. I believe this main point may be further refined in the discussion, also drawing on quantitative data from the existing literature.

VERSION 2 - AUTHOR RESPONSE

Reviewer: 2

- **Only a minor edit in the abstract line 13. Please change "one of the worst areas in UK due to high cancer incidence" to "UK areas with disproportionately high cancer incidence"**

The abstract has been completely reformatted.

Reviewer: 3

- **As the authors pointed out, numerical/quantitative statistics have not been performed in the review. It would have been interesting, though to get some more detail on the quantitative findings those studies, which include quantitative statistics. This would be important for the discussion regarding the kind of research and methods that need to be undertaken for a better understanding of the subject.**
- **In the same regard, the quantitative results of the spatially explicit models should be presented briefly. This could lead to a discussion on which methods may be employed in further studies (e.g. Besag-Newell, Bayesian Besag-York-Mollié, spatial scan statistics or other statistical methods for areal-cross-sectional data).**

As these two suggestions are related we will address them together. We included in the new Supplementary File 4 a table with numerical results. We focused the numerical data extraction on the studies applying spatially explicit methods. We highlighted in the Discussion section that this illustrates the heterogeneity in our included studies (Page 13, lines 346-349).

The numerical results of interest were: number of cases analysed, age group considered, period of study (years), outcomes (e.g. incidence rates, standardised incidence ratios, relative risks), if there was any adjustment for covariates, and in case of model comparisons, the method used and its respective values.

We hope this helps to clarify that even when considering a spatially explicit approach, the objectives are different, as well as cancer types, age groups, period considered, geographic location and scale, methods, and outcomes. In light of the above-explained reasons, we included some numerical results in our supplementary files, but we maintained the focus of a narrative synthesis for summarizing the existing evidence.

- **The authors discuss only the work of Arcaya et al., which reviews a similar subject for the United States. Here, too, one main limitation is the lack of publications applying spatial statistical models. It would be interesting to learn whether such research has been performed successfully elsewhere.**

In our Discussion section, we had previously cited other reviews in addition to Arcaya et al., such as the literature review by Gomez et al (2015). and the comprehensive review by Mihor et al. (2020).

In this new version, we bring back to the Discussion section two studies, originally cited only in the Introduction section: The systematized review by Roquette, Painho and Nunes (2017), and the systematic review by Zahnd and McLafferty (2017) (page 15, lines 415-436). In addition, we incorporated into our Discussion section two other systematic reviews by Thompson et al. (2019), and El Khoury (2024), and one scoping review by Bizuayehu et al. (2023) (Pages 15-16, lines 437-480).

These new references were conducted by retrieving studies from different countries, periods, cancer types, and accounting for different measurements (incidence, mortality, survival). They also highlighted the importance and the need for more cancer epidemiology research considering spatially explicit approaches.
